# Dual inhibition of cell cycle progression and apoptotic resistance in breast cancer by novel benzimidazole-based therapeutics

**DOI:** 10.1007/s00210-026-05206-y

**Published:** 2026-03-18

**Authors:** Murat Keser, Hakan Akgün, Ferdi Oguz, Harika Atmaca, Hakan Bektaş, Emre Menteşe, Canan Albay, Suleyman Ilhan

**Affiliations:** 1 Department of Medical Oncology, Medicana International İzmir Hospital, İzmir, Türkiye; 2 Department of Chemistry, Faculty of Science and Art, Giresun University, Giresun, Türkiye; 3 Graduate School of Health Sciences, Cellular and Molecular Medicine, Koç University, Istanbul, Türkiye; 4 Department of Biology, Faculty of Engineering and Natural Sciences, Celal Bayar University, 45140 Manisa, Turkey; 5 Department of Chemistry, Faculty of Science and Art, Recep Tayyip Erdogan University, Rize, Türkiye

**Keywords:** Benzimidazole, Breast cancer, Apoptosis, Cell cycle, Cell signaling

## Abstract

Breast cancer remains a leading cause of cancer-related mortality among women worldwide, underscoring the urgent need for more effective and targeted therapeutic strategies. Due to their diverse biological activities and structural flexibility, Benzimidazole derivatives have emerged as promising anticancer candidates. This study focuses on the development and chemical preparation of novel benzimidazole-based analogs, followed by an evaluation of their growth-inhibitory effects on MCF-7 and MDA-MB-231 human breast cancer cell lines. Among the synthesized molecules, compound 8a exhibited the most potent and selective anti-proliferative effect, with significantly lower IC₅₀ values in cancer cells compared to normal cells. Flow cytometric analysis revealed that 8a induced apoptosis and caused an arrest in the cell division process during the G2/M transition phase. *In silico* interaction analysis further indicated a strong binding affinity of compound 8a to key cell cycle and apoptosis-regulating proteins, particularly Cyclin E, CDK2, Bcl-2, and Bcl-xL. These findings indicate that compound 8a exerts a dual mechanism of action by modulating both proliferative and survival pathways, highlighting its potential as a promising lead candidate for anticancer drug development.

**Supplementary Information:**

The online version contains supplementary material available at 10.1007/s00210-026-05206-y.

## Introduction

Even with advancements in diagnostic and therapeutic approaches, breast cancer retains its position as a significant worldwide health challenge and ranks second in cancer-related mortality among the female population. Conventional chemotherapeutic agents, particularly platinum-based drugs such as cisplatin, carboplatin, and oxaliplatin, are limited by poor tumor selectivity, substantial systemic toxicity, and the frequent emergence of multidrug resistance. Given these shortcomings, a compelling rationale exists for pursuing new pharmacological strategies that more effectively target cancer cells while reducing harm to healthy tissues (Bustos et al. 2022; Çakır et al. 2024).

In this regard, bioactive organic molecules have emerged as promising alternatives to conventional therapies. Heterocyclic structures, particularly benzimidazole scaffolds, have gained prominence due to their versatile biological functions and structural adaptability. Benzimidazoles have shown potential in a wide array of pharmacological activities, including anti-inflammatory (Ahmed Abdel-Mohsen et al. 2016), antiviral (Tonelli et al. 2010; Sharma et al. 2009a), antibacterial (He et al. 2004; Göker et al. 2005), antifungal (Sharma et al. 2009b; Vinoth Kumar et al. 2013; Can et al. 2017), antioxidant (Özil et al. 2018; Taha et al. 2024), and notably, antiproliferative and anticancer properties (Nashaat et al. 2020).

The benzimidazole scaffold, consisting of fused benzene and imidazole rings, provides high electron density and multiple interaction sites, enabling strong interactions with diverse intracellular targets. This privileged scaffold is thus frequently employed in the design of innovative anticancer compounds with superior pharmacological profiles (Tahlan et al. 2019).

Although numerous benzimidazole derivatives have been reported as anticancer agents, the majority of previous studies have primarily focused on general cytotoxicity screening or single-pathway targeting, often emphasizing either cell cycle disruption or apoptosis induction alone. In addition, many reported compounds lack mechanistic integration between experimental and computational findings, limiting their translational relevance. In contrast, the present study integrates in vitro cytotoxicity, cell cycle analysis, apoptosis assessment, and in silico interaction studies within a unified experimental framework. Importantly, our findings suggest a dual inhibitory effect involving both cell cycle progression and apoptotic resistance, supported by concordant biological and docking data. This combined strategy enables a more comprehensive assessment of structure–activity relationships and provides mechanistic insight beyond conventional antiproliferative screening. In this context, the molecular design of the investigated benzimidazole derivatives was guided by the dual objective of simultaneously targeting cell cycle dysregulation and apoptotic resistance, two interconnected hallmarks of breast cancer chemoresistance. The benzimidazole core was selected as a privileged scaffold due to its reported ability to interact with ATP-binding sites of cyclin-dependent kinases as well as hydrophobic grooves of anti-apoptotic Bcl-2 family proteins. Structurally, the planar aromatic nature of the benzimidazole ring enables π-π stacking and hydrogen bonding interactions within kinase active sites, while appropriate substitution patterns allow extension toward the BH3-binding region of apoptosis-regulating proteins. This design strategy integrates dual biological activities within a single chemical framework, providing a mechanistic basis for subsequent biological and computational investigations.

Building on these attributes and addressing gaps in previous benzimidazole-based anticancer studies, the present work aimed to synthesize novel benzimidazole derivatives and investigate not only their cytotoxic potential but also their underlying mechanisms of action, including cell cycle regulation and apoptotic signaling, to identify a promising lead compound for further anticancer development. Specifically, the study focuses on benzimidazole derivatives rationally designed to interfere with key regulators of cell cycle progression and apoptotic resistance, two hallmarks frequently associated with chemoresistance in breast cancer. By integrating experimental cytotoxicity and mechanistic assays with molecular docking against apoptosis- and cell cycle-related targets, this work aims to establish a mechanistically supported structure–activity relationship that goes beyond purely phenotypic antiproliferative evaluation.

## Materials and methods

### Synthesis

**Scheme 1:**
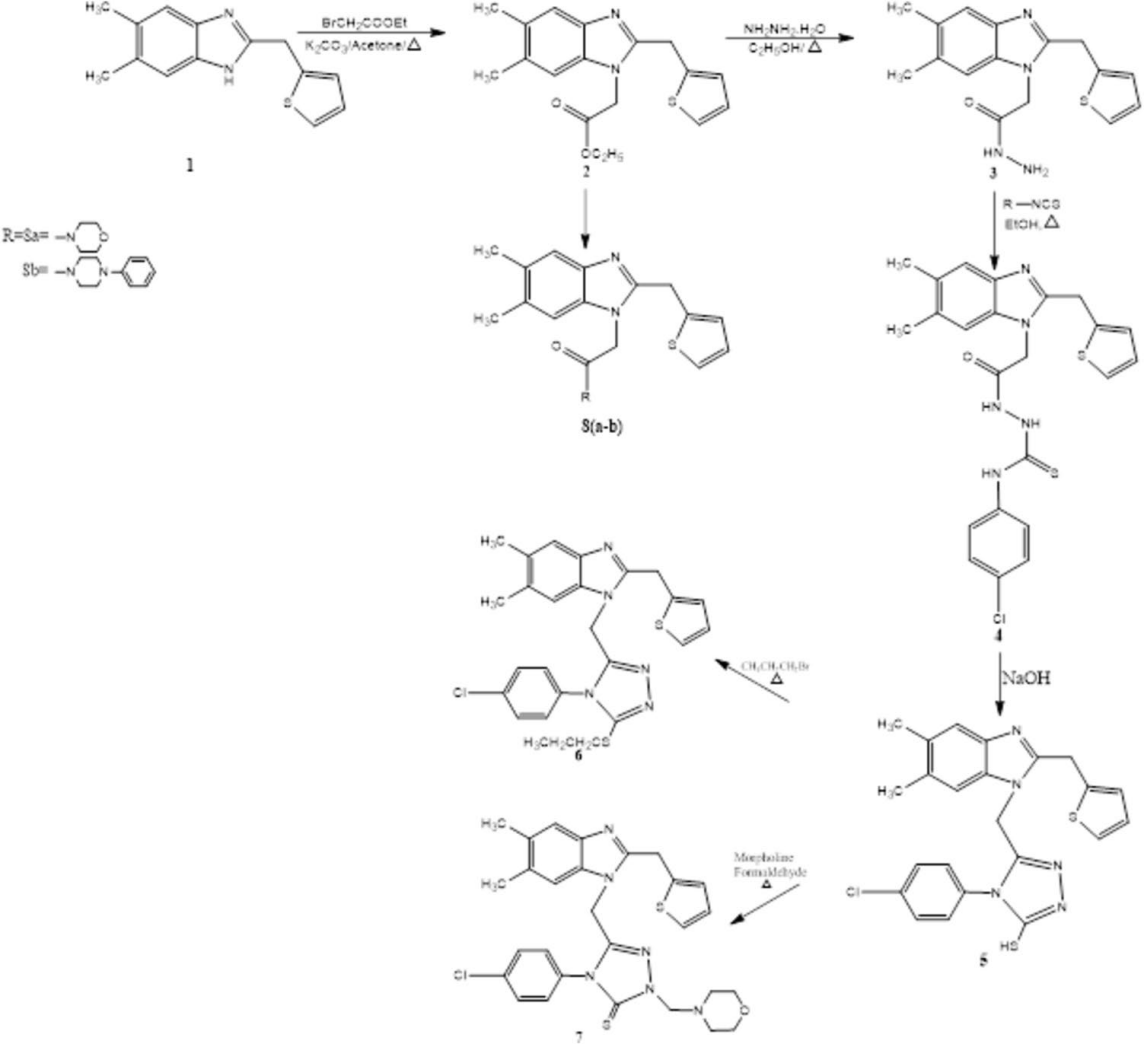
Synthetic route for the preparation of the target compounds

Structural characterization of the synthesized benzimidazole derivatives (Scheme 1) was performed using a Bruker AVANCE III 400 MHz NMR spectrometer, an Optimelt digital melting point apparatus, and an Agilent LC/MS-TOF mass spectrometer (Tahlan et al. 2019).

#### General procedure for synthesis of 5, 6-dimethyl-2-(2-thienylmethyl)−1H-benzimidazole (1)

Compound 1, serving as the starting material for our syntheses, was obtained as the product of the reaction between 4,5-dimethyl-o-phenylenediamine and the corresponding iminoester hydrochloride. This synthesis was carried out according to methods reported in the literature, and the structure was elucidated (Menteşe et al. 2018).

#### General procedure for synthesis of ethyl [5, 6-dimethyl-2-(2-thienylmethyl)−1H-benzimidazol-1-yl]acetate (2)

Compound 1 (0.01 mol) was refluxed with an equimolar amount of ethyl bromoacetate (0.01 mol) in 30 mL of acetone in a 50 mL round-bottom flask for 5 h. After completion, distilled water was added to the reaction mixture, and the resulting white precipitate was filtered. The crude product was purified by recrystallization from a 1:1 acetone–water mixture, dried under vacuum, and identified as compound 2. Yield: 3.02 g, 92%. Melting point: 120–121 °C. ^1^H-NMR (DMSO-d_6_) ppm: 0.29 (t, J = 7.1 Hz, 3H, CH_3_), 1.44 (s, 3H, CH_3_),1.65 (s, 3H, CH_3_), 3.18 (d, J = 7.1 Hz, 2H, CH_2_), 3.58 (s, 2H, CH_2_), 4.26 (s, 2H, CH_2_), 6.08 (m, 2H, Ar–H), 6.36 (s, 1H, Ar–H), 6.52 (m, 2H, Ar–H), ^13^C-NMR (DMSO-d_6_) ppm: 14.41 (CH_3_), 20.55 (2CH_3_), 28.03 (CH_2_), 44.92 (CH_2_), 61.58 (CH_2_), ArC: [110.71 (C), 119.33 (C), 125.77 (C), 126.71 (C), 127.12 (C), 130.38 (C), 131.13 (C), 135.11 (C), 138.20 (C), 141.18 (C)], 152.50 (C = N), 168.30 (C = O). LC/TOF–MS, *m*/*z*: 329.1362 [M + H] + (Ilhan et al. 2023b).

#### General procedure for the synthesis of 2-[5, 6-dimethyl-2-(2-thienylmethyl)−1H-benzimidazol-1-yl]acetohydrazide (3)

Compound 2 (0.01 mol) was refluxed with hydrazine hydrate (0.04 mol) and 20 mL of absolute ethanol in a 50 mL round-bottom flask for 5 h. The reaction progress was monitored by TLC and confirmed to be complete. The mixture was then cooled and kept at 0 °C overnight in a deep freezer. After complete precipitation, the solid was filtered, purified with ethanol, and dried under vacuum. The resulting pure product was identified as compound 3. Yield: 2.51 g, 79%. Melting point: 253–254 °C. ^1^H-NMR (DMSO-d_6_) ppm: 1.44 (s, 3H, CH_3_), 1.66 (s, 3H, CH_3_), 3.58 (s, 2H, NCH_2_), 3.89 (s, 2H, CH_2_), 6,09–6.13 (m, 2H, Ar–H), 6.33 (s, 1H, Ar–H), 6.48 (s, 1H, Ar–H), 6.52–6.54 (m, 2H, Ar–H), 8.63 (s, 1H, NH), ^13^C-NMR (DMSO-d_6_) ppm: 20.54 (2CH_3_), 28.51 (CH_2_), 44.85 (CH_2_), ArC: [110.62 (C), 119.29 (C), 125.63 (C), 126.58 (C), 127.23 (C), 130.20 (C), 130.88 (C), 134.52 (C), 139.36 (C), 141.07 (C)], 152.76 (C = N), 166.47 (C = O). LC/TOF–MS, *m*/*z*: 315.1320 [M + H] +.

#### General procedure for the synthesis of N-(4-chlorophenyl)−2-{[5,6-dimethyl-2-(2-thienylmethyl)−1H-benzimidazol-1-yl]acetyl}hydrazinecarbothioamide (4)

Compound 3 (0.01 mol) and p-chlorophenyl isothiocyanate (0.01 mol) were refluxed in 40 mL of absolute ethanol in a 100 mL round-bottom flask for 10 h. The reaction progress was monitored by TLC and confirmed complete. The resulting precipitate was filtered, purified with ethanol, and dried under vacuum. The purified product was identified as compound 4. Yield: 4.16 g, 86%. Melting point: 166–167 °C. ^1^H-NMR (DMSO-d_6_) ppm: 1.44 (s, 3H, CH_3_), 1.65 (s, 3H, CH_3_), 3.59 (s, 2H, CH_2_), 3.89(s, H, NH), 4.08 (s, 2H, CH_2_), 6.11 (m, 2H, Ar–H), 6.34 (s, 1H, Ar- H), 6.53–6.61 (m, 6H, Ar–H), 8.97 (s, H, NH), 9.64 (s, 1H, NH), ^13^C-NMR (DMSO-d_6_) ppm: 20.32 (CH_3_), 20.55 (CH_3_), 28.14 (CH_2_), 44.93 (CH_2_), ArC: [110.66 (C), 119.32 (C), 125.61 (C), 126.60 (2C), 127.25 (2C), 128.63 (2C), 130.35 (C), 131.01 (C), 134.65 (C), 138.48 (C), 139.32 (C), 141.10 (2C)], 152.74 (C = N), 167.01 (C = O), 181.20 (C = S). LC/TOF–MS, *m*/*z*: 484.1015 [M] +.

#### General procedure for the synthesis of 4-(4-chlorophenyl)−5-{[5, 6-dimethyl-2-(2-thienylmethyl)−1H-benzimidazol-1-yl]methyl}−4H-1,2,4-triazole-3-thiol (5)

A solution of compound 4 (0.01 mol) in absolute ethanol was treated with an aqueous solution of sodium hydroxide (0.01 mol), and the mixture was refluxed for 4.5 h. After cooling to room temperature, the reaction mixture was poured into cold water, and the pH was adjusted to 4 using dilute hydrochloric acid. The resulting mixture was kept in a freezer. The precipitate formed was recrystallized from an alcohol–water mixture, dried under vacuum, and identified as compound 5. Yield: 3.02 g, 70%. Melting point: 227–228 °C. ^1^H-NMR (DMSO-d_6_) ppm: 1.44 (s, 3H, CH_3_), 1.65 (s, 3H, CH_3_), 3.45- 3.63 (bs, 2H, CH_2_), 4.13- 4.47 (bs, 2H, CH_2_), 5.98, 6.10 (m, 3H, Ar–H), 6.45–6.70 (m, 6H, Ar–H), 13.04 (s, 1H, SH), ^13^C-NMR (DMSO-d_6_) ppm: 20.27 (CH_3_), 20.49 (CH_3_), 27,87 (CH_2_), 27.92 (CH_2_), ArC: [103.50 (C), 110.75 (C), 118.99 (C), 125.80 (C), 126.68 (C), 127.12 (2C), 127.37 (C), 129.94 (2C), 130.48 (2C), 132.18 (C), 135.00 (C), 135.36 (2C), 148.04 (C), 152.06 (C)], 184.86(C = N). (LC/TOF–MS, *m*/*z*: 466.0912 [M] +.

#### General procedure for the synthesis of 1-{[4-(4-chlorophenyl)−5-(propylthio)−4H-1,2,4-triazol-3-yl]methyl}−5,6-dimethyl-2-(2-thienylmethyl)−1H-benzimidazole (6)

To compound 5 (0.01 mol), a solution of potassium carbonate (0.02 mol) in 30 mL of acetone was added. The reaction mixture was stirred at room temperature for 1 h, followed by the addition of 1-bromopropane (0.01 mol). The solution was stirred at room temperature for 10 h. After completion, distilled water was added, and the precipitate was filtered. The product was recrystallized from an alcohol–water mixture, dried under vacuum, and identified as compound 6. Yield: 4.06 g, 80%. Melting point: 207–208 °C. ^1^H-NMR (DMSO-d_6_) ppm: 0.18–0.21 (m, 3H, CH_3_), 0.74–0.79 (m, 2H, CH_2_), 2.16–2.19 (m, 2H, CH_2_), 1.40 (m, 3H, CH_3_), 1.66 (m, 3H, CH_3_), 3.40 (s, 2H, CH_2_), 4.60 (s, 2H, CH_2_), 5.96 (s, 1H, Ar–H), 6.06 (d, J = 4.3 Hz, 1H, Ar–H), 6.30 (d, J = 8.4 Hz, 1H, Ar–H), 6.34 (s, 2H, Ar–H), 6.41 (s, 1H, Ar–H), 6.51 (s, 1H, Ar- H), 6.60 (d, J = 7.6 Hz, 2H, Ar–H), ^13^C-NMR (DMSO-d_6_) ppm: 13.30 (CH_3_), 13.32 (CH_3_), 20.26 (CH_3_), 22.76 (CH_2_), 28.04 (CH_2_), 34.56 (CH_2_), 38.56 (CH_2_), ArC: [110.46 (C), 119.17 (C), 125.71 (C), 126.56 (C), 127.12 (C), 129.48 (2C), 130.23 (2C), 130.84 (C), 131.42 (C), 133.48 (C), 135.51(2C), 138.27 (2C), 140.74 (C), 151.96 (C), 152.22 (C)]. LC/TOF–MS, *m*/*z*: 508.1363 [M] +.

#### General procedure for the synthesis of 4-(4-chlorophenyl)−5-{[5, 6-dimethyl-2-(2-thienylmethyl)−1*H*-benzimidazol-1-yl]methyl}−2-(morpholin-4-ylmethyl)−2,4-dihydro-3*H*−1,2,4-triazole-3-thione (7).

To compound 5 (0.01 mol), equimolar amounts of morpholine and formaldehyde were added. Then, 30 mL of dichloromethane was added to the mixture, which was stirred at room temperature for 8 h. The solvent was evaporated, diethyl ether was added to the residue, and the resulting solid was filtered and dried under vacuum. The purified product was identified as compound 7. Yield: 4.29 g, 76%. Melting point: 150–151 °C. ^1^H-NMR (DMSO-d_6_) ppm: 1.40- 1.44 (m, 3H, CH_3_), 1.66 (bs, 3H, CH_3_), 1.80 (m, 4H, 2CH_2_) 2.70 (bs, 4H, 2CH_2_), 3.37 (bs, 2H, CH_2_), 4.14 (bs, 2H, CH_2_), 4.55 (bs, 2H, CH_2_), 5.98–6.09 (bm, 4H, Ar–H), 6.40–6.51 (bm, 3H, Ar–H), 6.66–6.67 (bm, 2H, Ar–H),^13^C-NMR (DMSO-d_6_) ppm: 20.27 (CH_3_), 20.43 (CH_3_), 27.94 (2CH_2_), 50.59 (2CH_2_), 66.56 (2CH_2_), 69.29 (CH_2_), ArC: [110.69 (C), 119.32 (C), 125.73 (C), 126.60 (C), 127.14 (C), 129.99 (2C), 131.12 (2C), 132.58 (2C), 134.12 (2C), 136.16 (C),138.63 (C), 140.82 (C), 141.02 (C), 146.77 (C), 151.98 (C)]. LC/TOF–MS, *m*/*z*: 565.1547[M] +.

#### General procedure for the synthesis of 5, 6-dimethyl-1-(2-morpholin-4-yl-2-oxoethyl)−2-(2-thienylmethyl)−1H-benzimidazole (8a)

To compound 2 (0.01 mol), 3 molar equivalents of morpholine were added. The reaction mixture was refluxed over asbestos wire for 24 h. After completion, the cooled solution was poured into ice-cold water. The aqueous layer was extracted with chloroform. After evaporation of chloroform, the resulting solid was recrystallized from an ethyl acetate–diethyl ether mixture, dried under vacuum, and identified as compound 8a. Yield: 2.66 g, 72%. Melting point: 187–188 °C. ^1^H-NMR (DMSO-d_6_) ppm: 1.44 (s, 3H, CH_3_), 1.66 (s, 3H, CH_3_), 2.29–2.31 (bs, 2H, CH_2_), 2.72–2.83 (bs, 4H, 2CH_2_), 3.49–3,54 (bm, 2H, CH_2_), 3.94 (bs, 2H, CH_2_), 4.29 (bs, 2H, CH_2_), 6.10–6.11 (bm, 2H, Ar–H),6.32 (d, J = 3.2 Hz, 1H, Ar–H), 6.48–6.53 (m, 2H, Ar–H), ^13^C-NMR (DMSO-d_6_) ppm: 20.29 (CH_3_), 20.49 (CH_3_), 28.20 (CH_2_), 42.39 (CH_2_), 44.62 (CH_2_), 45.18 (CH_2_), 60.12 (CH_2_), 66.41 (CH_2_), ArC: [110.74 (C), 119.26 (C), 125.56 (C), 126.69 (C), 127.12 (C), 129.90 (C), 130.63 (C), 135,57 (C) 139.31 (C), 141.10 (C)], 152.88 (C = N), 166.47 (C = O). LC/TOF–MS, *m*/*z*: 370.1610 [M + H] +.

#### General procedure for the synthesis of 5, 6-dimethyl-1-[2-oxo-2-(4-phenylpiperazin-1-yl)ethyl]−2-(2-thienylmethyl)−1H-benzimidazole (8b)

To compound 2 (0.01 mol), 3 molar equivalents of 4-phenylpiperazine were added. The reaction mixture was refluxed over the asbestos wire for 24 h. After completion, the cooled solution was poured into ice-cold water. The aqueous phase was extracted with chloroform. Following evaporation of chloroform, the obtained solid was recrystallized from an ethyl acetate–diethyl ether mixture, dried under vacuum, and identified as compound 8b. Yield: 3.11 g, 70%. Melting point: 207–208 °C. ^1^H-NMR (DMSO-d_6_) ppm: 1.55–1.56 (m, 6H, 2CH_3_), 1.88 (bs, 4H, 2CH_2_), 2.08 (bs, 4H, 2CH_2_), 2.51 (bs, 4H, 2CH_2_), 5.81 (t, J = 6.9 Hz, 2H, Ar–H), 5.96 (d, J = 7.5 Hz, 4H, Ar–H), 6.25 (t, J = 7.8 Hz, 4H, Ar–H), ^13^C-NMR (DMSO-d_6_) ppm: 20.26 (CH_3_), 20.39 (CH_3_), 28.25 (CH_2_), 42.33 (CH_2_), 44.60 (CH_2_), 45.20 (CH_2_), 60.15 (CH_2_), 66.40 (CH_2_), ArC: [110.75 (C), 119.22 (C), 125.55 (C), 126.60 (C), 127.25 (2C), 129.98 (C), 130.66 (2C), 132.16 (2C), 134.50 (C), 136.82 (C), 139.34 (C), 141.12 (C), 143.16 (C)], 152.78 (C = N), 165.45 (C = O) LC/TOF–MS, *m*/*z*: 445.2073 [M + H] +.

### Cell culture

The human breast carcinoma cell lines MCF-7 and MDA-MB-231 were procured from ATCC (USA), and the MCF-10A mammary epithelial cell line, which is non-tumorigenic, was provided by the Health Protection Agency (UK). Cell lines were selected based on their distinct molecular and phenotypic characteristics. MCF-7 cells represent a hormone receptor–positive, less aggressive breast cancer subtype, whereas MDA-MB-231 cells are a triple-negative, highly invasive model associated with poor prognosis and therapeutic resistance. The use of these two complementary breast cancer cell lines allowed the evaluation of compound activity across biologically diverse disease phenotypes. In addition, the non-tumorigenic MCF-10A breast epithelial cell line was included as a normal control to assess the selectivity and safety profile of the synthesized compounds. MCF-7 cells were propagated in RPMI-1640 medium, while MCF-10A cells were sustained in DMEM/F12 medium enriched with 10% fetal bovine serum (FBS), 1% L-glutamine, and 1% penicillin–streptomycin. All cultured cell populations were kept at 37 °C in a CO₂-incubated, humidity-controlled environment with 5% CO₂. MCF-7 and MDA-MB-231 cells were used between passages 6 and 18, while MCF-10A cells were used between passages 5 and 15 to minimize phenotypic drift. The culture media were routinely refreshed every 2–3 days, and cells were detached using 0.25% trypsin–EDTA upon reaching approximately 70–80% confluency (Ilhan et al. 2023a).

### MTT Assay for cell viability

Antiproliferative activity was evaluated using the MTT assay, which quantifies mitochondrial activity as an indicator of cell viability. Cells (approximately 1 × 10^4^ per well) were seeded into 96-well microplates with 100 µL of complete culture medium. Cells (approximately 1 × 10^4^ per well) were seeded into 96-well microplates with 100 µL of complete culture medium and allowed to attach for 24 h before treatment. After cell attachment, the medium was replaced with fresh medium containing varying concentrations of the test compounds for 24, 48, or 72 h, prepared from concentrated stock solutions dissolved in DMSO. The final DMSO concentration in all wells did not exceed 0.1% (v/v), and vehicle control groups were included in all experiments.The concentration ranges of the tested compounds were selected based on preliminary screening experiments and previously reported concentration ranges for benzimidazole-based anticancer agents. Initial broad-range screening was performed to identify cytotoxic and non-cytotoxic dose intervals, followed by finer concentration gradients to enable accurate IC₅₀ determination. All treatments were designed to include sub-cytotoxic and cytotoxic concentrations to allow reliable dose–response analysis.A stock solution of MTT (5 mg/mL) was prepared in phosphate-buffered saline (PBS), and 10 µL of this solution was added to each well to achieve a final concentration of 0.5 mg/mL. The plates were incubated for an additional 4 h at 37 °C to allow the formation of formazan crystals. Subsequently, the supernatant was gently removed, and the resulting formazan crystals were solubilized by adding 100 µL of dimethyl sulfoxide (DMSO) to each well. The absorbance of the dissolved product was then quantified at a wavelength of 570 nm using a spectrophotometric plate reader. The IC₅₀ values, representing the concentration at which 50% of cell viability was inhibited, were determined using Calcusyn (Biosoft) software for dose–response curve analysis (Ilhan et al. 2023a).

### Apoptosis evaluation by flow cytometry

To assess the pro-apoptotic potential of compound 8a, human breast cancer cells (1 × 10^6^) were plated in 6-well culture dishes and treated with its half-maximal inhibitory concentration (IC₅₀) for 72 h. After incubation, cells were gently harvested, washed twice with cold phosphate-buffered saline (PBS), and resuspended in a binding buffer suitable for apoptosis detection assays. Apoptotic cell populations were identified using FITC-conjugated Annexin V and propidium iodide (PI) dual staining. The stained samples were incubated at ambient temperature in the dark to prevent photo-degradation of the dyes. Flow cytometric analysis was subsequently performed to quantify the distribution of viable, early apoptotic, late apoptotic, and necrotic cells, based on membrane integrity and externalized phosphatidylserine levels (Ilhan et al. 2023b).

### Assessment of cell cycle distribution

The effect of compound 8a on cell cycle progression was evaluated by flow cytometry. Breast cancer cells (1 × 10^4^) were seeded in 6-well plates, treated with either the IC₅₀ concentration of the compound or DMSO as a control, and incubated for up to 72 h. Post-treatment, cells were fixed in 70% cold ethanol and stained with Muse Cell Cycle reagent. After a 30-min dark incubation, the quantification of cellular DNA content was performed using a benchtop flow cytometric system, in strict accordance with the manufacturer’s standardized protocol, to investigate cell cycle progression at the population level (Ilhan et al. 2023a).

### Molecular docking analysis

Molecular docking was conducted using Molegro Virtual Docker (version 6.0). The MolDock scoring function was employed to evaluate ligand–protein interactions. Docking results were expressed as MolDock Score values (arbitrary units), where more negative scores indicate stronger predicted binding.The target proteins selected for this analysis included Bcl-2 (PDB ID: 2W3L), Bcl-xL (1YSI), CDK2 (1HCK), and Cyclin E (1W98), all retrieved from the Protein Data Bank. The chemical structure of compound 8a was optimized using Gaussian 09 W and subsequently processed in Maestro using the LigPrep module under physiological pH conditions, applying the OPLS 2005 force field.In addition to compound 8a, well-established reference inhibitors were included for comparative docking analysis. ABT-199 and ABT-737 were selected as reference inhibitors for Bcl-2 and Bcl-xL, respectively, due to their clinically validated anti-apoptotic inhibition profiles. Roscovitine was employed as a reference inhibitor for CDK2 and Cyclin E, given its well-documented cyclin-dependent kinase inhibitory activity. Protein structures were prepared using Molegro, which involved the removal of crystallographic water molecules, the addition of polar hydrogens, the assignment of partial charges, and the correction of bond orders. Energy minimization was performed until the structures achieved a root-mean-square deviation (RMSD) threshold of 0.30 Å. A grid box with dimensions of 60 Å × 60 Å × 60 Å was defined and centered at X = 21.41, Y = 3.62, Z = 21.94. Docking simulations were carried out for all target proteins using these parameters. The docking protocol was validated by re-docking co-crystallized ligands, which produced RMSD values below 2 Å, indicating reliable accuracy (Atmaca et al. 2024).

### Statistical analysis

All statistical analyses, including half-maximal inhibitory concentrations (IC₅₀) calculations and graph generation, were conducted using GraphPad Prism 5 (GraphPad Software, La Jolla, CA, USA). Data from experiments performed in triplicate were analyzed using one-way ANOVA, followed by Dunnett’s post hoc test for multiple comparisons. A p-value of less than 0.05 was considered statistically significant.

## Results

### Anti-proliferative activities of the newly developed benzimidazole compounds

Cytotoxic activity of the synthesized benzimidazole derivatives was evaluated by determining IC₅₀ values using the MTT assay after 72 h of exposure in MCF-7 and MDA-MB-231 breast cancer cell lines, as well as the non-malignant MCF-10A line (Table 1). Compound 8a exhibited the most potent anticancer activity with IC₅₀ values of 15.5 ± 2.2 µM in MCF-7 and 14.3 ± 0.8 µM in MDA-MB-231 cells. Notably, this compound showed a higher IC₅₀ in non-tumorigenic MCF-10A cells (19.6 ± 0.6 µM), suggesting selectivity toward malignant cells. In contrast, compound 2 displayed poor cytotoxicity across all cell lines, with IC₅₀ values exceeding 150 µM. Several other derivatives, including compounds 3, 4, and 7, demonstrated intermediate cytotoxic profiles, with IC₅₀ values ranging from 62.4 ± 1.4 µM to 124.4 ± 1.8 µM. Compound 8b showed moderate activity against cancer cells but lacked the selectivity observed with 8a.

**Table 1:** The IC_50_ values (µM) of the synthesized compounds in MCF-7 and MDA-MB-231 breast cancer cells and MCF-10A non-tumorigenic breast cells after 72 h

	IC_50_ (µM)		
Compound	MCF-7	MDA-MB-231	MCF-10A
2	152.9 ± 1.2	189.6 ± 0.6	198.4 ± 0.8
3	124.4 ± 1.8	120.3 ± 2.2	108.7 ± 1.2
4	90.5 ± 1.4	103.6 ± 1.0	115.2 ± 1.6
5	116.6 ± 2.0	130.2 ± 2.2	150.6 ± 1.1
6	102.8 ± 1.5	130.7 ± 2.8	124.0 ± 2.2
7	62.4 ± 1.4	64.3 ± 0.6	89.4 ± 1.0
8a	15.5 ± 2.2	14.3 ± 0.8	19.6 ± 0.6
8b	88.9 ± 3.1	128.9 ± 1.0	112.6 ± 3.0
Cisplatin	18.8 ± 0.2	17.4 ± 0.4	18.1 ± 1.0

The IC₅₀ values were calculated from dose–response curves generated using nonlinear regression analysis of cell viability versus logarithmic compound concentration. Percentage cell viability values obtained from the MTT assay were plotted against compound concentrations, and IC₅₀ values were determined using the CalcuSyn software based on a sigmoidal dose–response model. To support the reliability of the IC₅₀ determination, dose–response curves for compound 8a in MCF-7, MDA-MB-231, and MCF-10A cell lines are provided in the Supplementary Information (Suppl. Fig. S1).These results position compound 8a as a lead candidate for further development due to its potent and selective anti-proliferative effects.

### Apoptotic induction by compound 8a

**Fig. 1:**
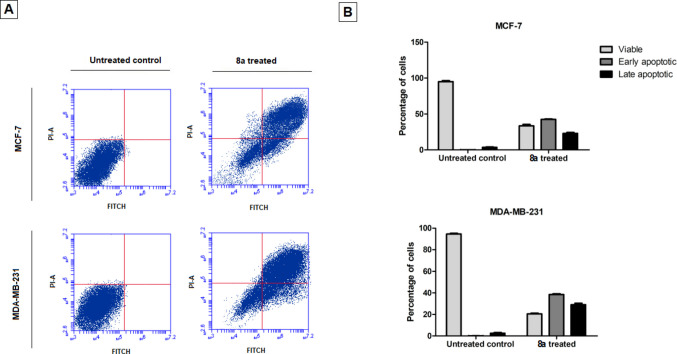
Flow cytometric analysis of apoptosis induced by compound 8a in MCF-7 and MDA-MB-231. **a** Representative dot plots showing the distribution of live (Annexin V⁻/PI⁻), early apoptotic (Annexin V⁺/PI⁻), late apoptotic (Annexin V⁺/PI⁺), and necrotic (Annexin V⁻/PI⁺) cells after treatment with compound 8a at IC₅₀ concentration for 72 h. **b** Quantitative analysis of apoptosis based on Annexin V-FITC/PI staining. Data represent the mean percentages of cells in each quadrant from three independent experiments. Asterisks indicate statistically significant differences compared to the untreated control group (*p < 0.05, **p < 0.01)

The ability of compound 8a to induce apoptosis was confirmed via flow cytometry. Treatment led to a significant shift in cell populations from viable to apoptotic phases in both MCF-7 and MDA-MB-231 cells. In MCF-7, viable cell percentage dropped from 95 to 33% after treatment, while early and late apoptotic cells increased to 42.5% and 23%, respectively. Similarly, in MDA-MB-231 cells, viability declined from 94% to 20.5%, with early apoptosis rising to 38.5% and late apoptosis to 29%. These observations support the pro-apoptotic effect of compound 8a (Fig. 1).

### Cell cycle disruption at the G2/M transition

**Fig. 2:**
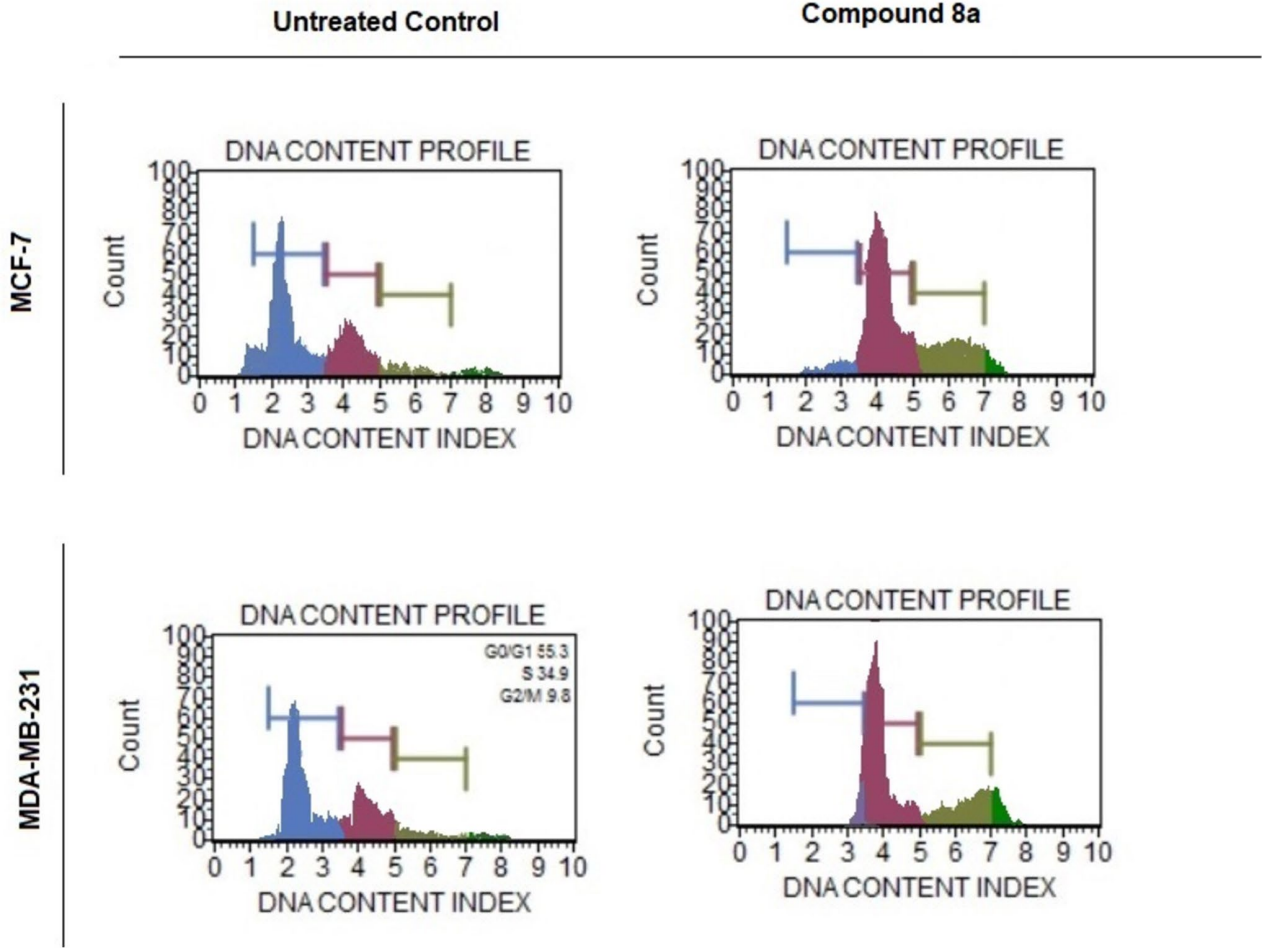
Cell cycle disruption induced by compound 8a in MCF-7 and MDA-MB-231 cells.. Flow cytometric analysis demonstrated that compound 8a significantly disrupted normal cell cycle progression in breast cancer cell lines. After treatment with compound 8a at its IC₅₀ concentration for 72 h, a marked accumulation of cells in the G₂/M phase was observed in both MCF-7 and MDA-MB-231 cells, accompanied by a corresponding reduction in the G₀/G₁ population. These findings suggest that the antiproliferative effect of compound 8a is associated with its ability to induce cell cycle arrest at the G₂/M checkpoint

Flow cytometric analysis demonstrated that compound 8a notably interfered with normal cell cycle progression. In both MCF-7 and MDA-MB-231 breast cancer models, a marked increase in the proportion of cells at the G2/M phase was observed after 72 h of treatment, accompanied by a decline in the G0/G1 population (Fig. 2). These findings imply that the compound's anti-proliferative effects could be linked to its capacity to trigger cell cycle arrest at the G2/M transition. Moreover, the effect became more pronounced with extended exposure, indicating a time-dependent mechanism of action.

### Molecular docking analysis

**Fig. 3:**
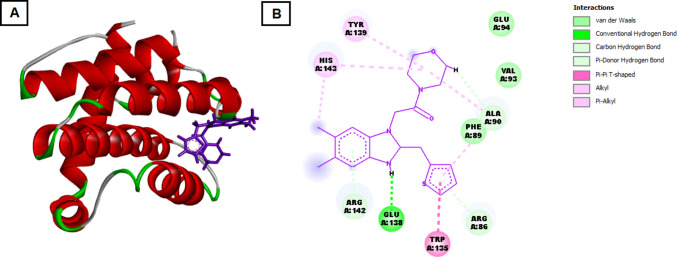
Binding interactions of compound 8a with the anti-apoptotic protein Bcl-2.. Molecular docking analysis revealed hydrogen bonding interactions between compound 8a and key residues of Bcl-2, including TYR139, HIS143, GLU94, and ARG142. The docking pose illustrates the positioning of the ligand within the Bcl-2 binding pocket, stabilized through polar contacts and hydrogen bonds. These interactions suggest a strong and specific affinity of compound 8a towards Bcl-2, potentially contributing to its pro-apoptotic activity

**Fig. 4:**
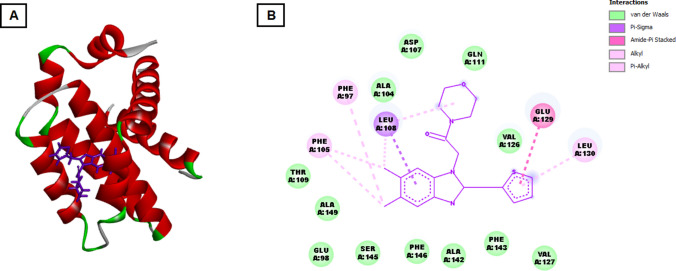
Docking interactions of compound 8a with Bcl-xL.. The docking pose of compound 8a in the Bcl-xL binding site shows predominantly hydrophobic and polar interactions. Key residues involved in ligand stabilization include VAL127, PHE105, and SER145. These interactions suggest that compound 8a can effectively engage with the Bcl-xL protein through non-covalent contacts, which may interfere with its anti-apoptotic function

**Fig. 5:**
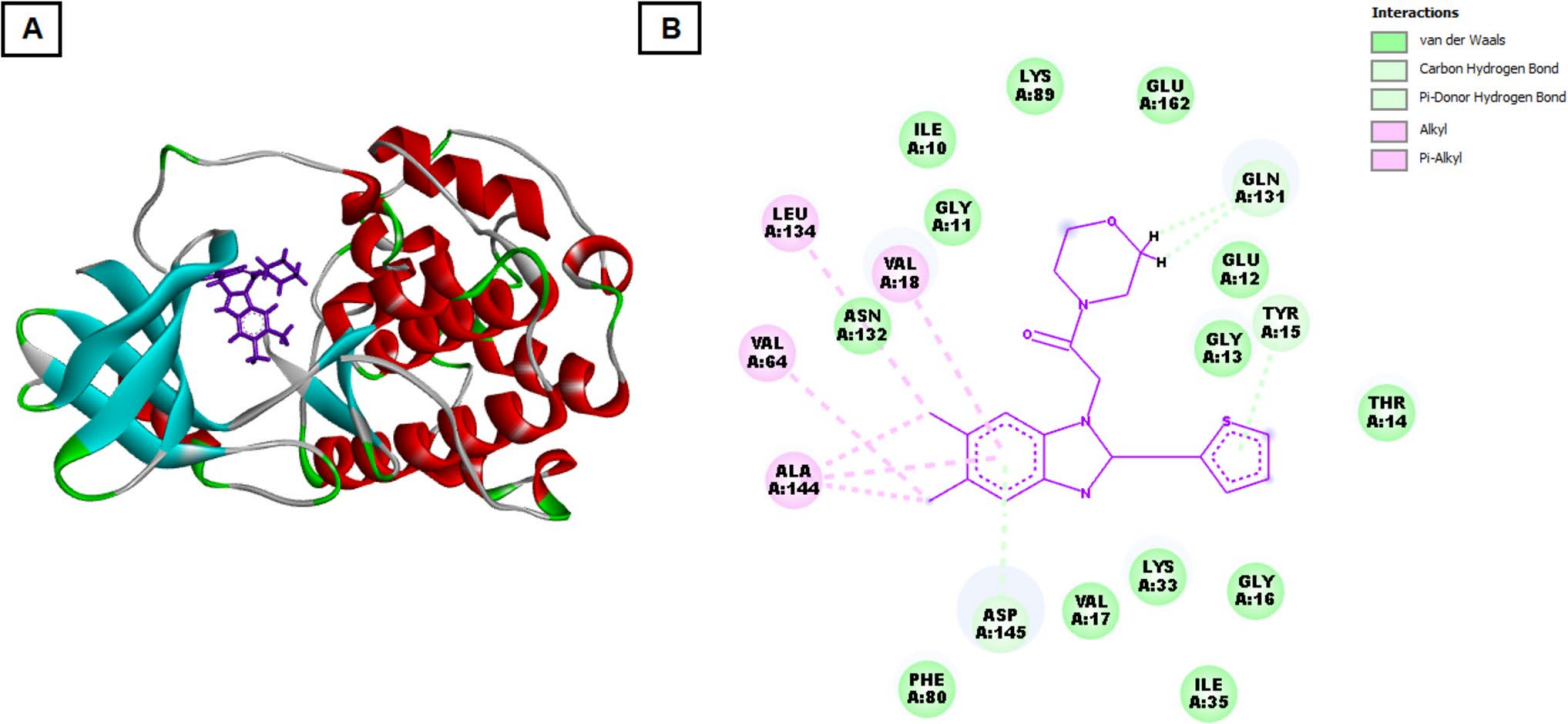
Molecular docking interaction of compound 8a with CDK2.. Docked pose of compound 8a in the active site of CDK2, showing key interactions with amino acid residues including VAL18, GLY11, and LYS33. These contacts contribute to the compound’s high binding affinity, highlighting its potential inhibitory effect on CDK2-mediated cell cycle regulation

**Fig. 6:**
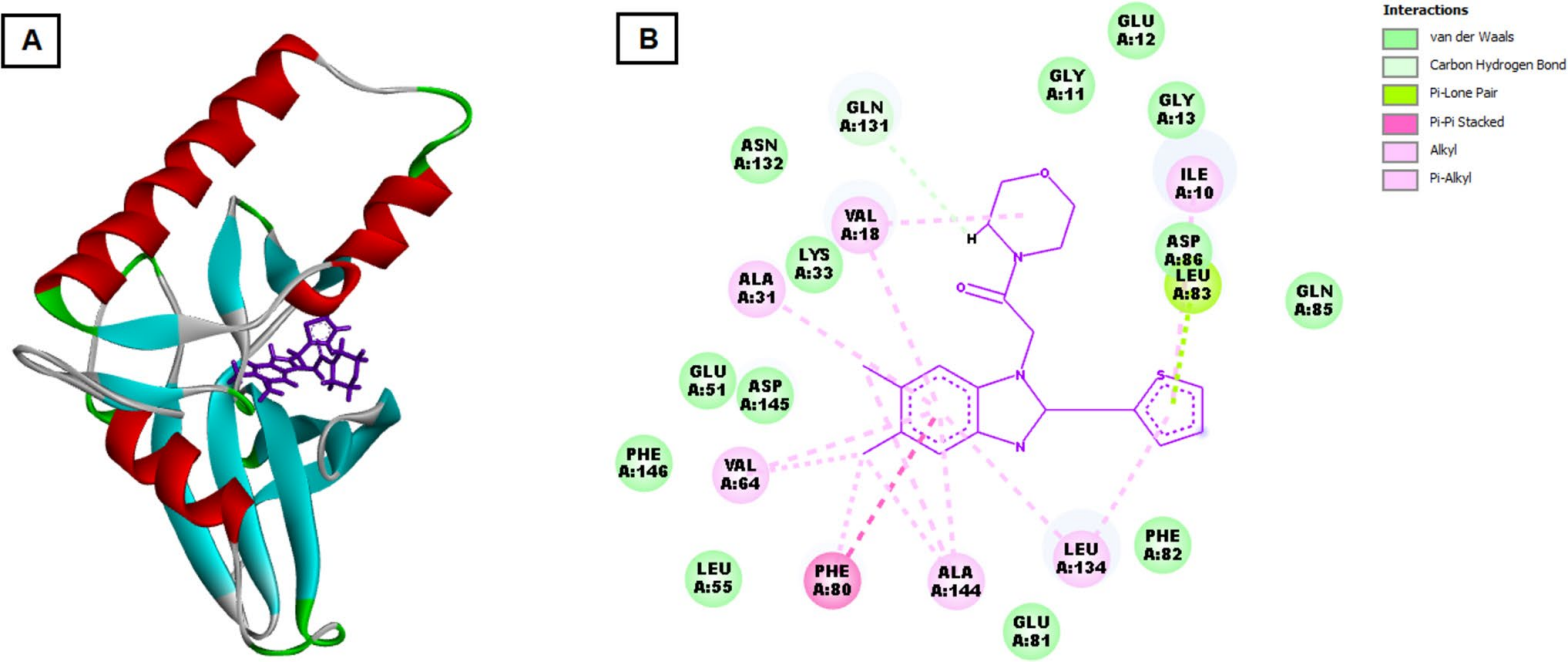
Molecular docking interaction of compound 8a with Cyclin E.. Binding mode of compound 8a within the active site of Cyclin E, displaying an extensive interaction network with residues such as GLN131, VAL18, and GLU12. These interactions support the highest observed binding affinity among the studied targets, indicating Cyclin E as a primary molecular target for compound 8a

Binding affinity was evaluated based on MolDock scores, where more negative values indicate stronger predicted interactions.Docking analysis revealed that compound 8a exhibited the highest binding affinity towards Cyclin E, with a docking score of –146.952 kcal/mol, followed by CDK2 (–135.259 kcal/mol), Bcl-xL (–131.800 kcal/mol), and Bcl-2 (–122.539 kcal/mol) (Table 2). Hydrogen bonding was explicitly observed in the Bcl-2 complex (Fig. 3), where interactions were formed with key residues such as TYR139, HIS143, GLU94, and ARG142. Bcl-xL binding involved predominantly hydrophobic and polar interactions with amino acids, including VAL127, PHE105, and SER145 (Fig. 4). In the CDK2 complex, significant contacts were observed with residues such as VAL18, GLY11, and LYS33 (Fig. 5). The Cyclin E-compound 8a complex demonstrated extensive interaction networks involving GLN131, VAL18, and GLU12, supporting a high binding affinity (Fig. 6). Since MolDock scores are not expressed as binding free energy values, estimated Ki values were not calculated.These findings suggest that compound 8a potentially targets key cell cycle progression and apoptosis regulators, with a strong interaction profile, particularly toward Cyclin E and CDK2, making it a promising lead candidate for further anticancer drug development.

**Table 2:** Molecular docking results of compound 8a and reference inhibitors obtained using Molegro Virtual Docker. Binding affinity was evaluated based on MolDock scores (arbitrary units), where more negative values indicate stronger predicted interactions. Hydrogen bond distances are reported in Å. Bold values indicate stronger binding interactions of compound 8a compared to the corresponding reference inhibitor for the same target protein. Since Molegro Virtual Docker employs a scoring-based docking approach rather than free energy–based calculations, binding affinity comparisons were performed using MolDock score values instead of estimated Ki values

Receptor (PDB ID)	Ligand/Inhibitor	MolDock score (a.u)	H Bond	Key interacting residues
Bcl-2 (2W3L)	8a	−122.539	−1.986	TYR139, HIS143, GLU94, VAL93, ALA90, PHE89, ARG86, ARG142, TRP135, GLU138
Bcl-2 (2W3L)	ABT-199	−110.543	−2.567	VAL93, GLU94, TYR139, ALA90, THR91, GLY87, HIS143, GLU138, ARG142, LEU134, ARG86, PHA89, TRP135
Bcl-xL (1YSI)	8a	−131.800	-	VAL127, PHE97, PHE105, PHE143, PHE146, ALA104, ALA142, ALA149, SER145, GLU98, THR109, LEU108, ASP107, GLN111, VAL126, VAL127, GLU129
Bcl-xL (1YSI)	ABT-737	−131.721	−0.419	ARG102, ARG100, TYR101, ALA104, ARG103, ASP107, VAL126, LEU130, PHE105, GLU98, SER145, PHE97, PHE146, LEU108, ALA149
CDK2 (1HCK)	8a	−135.259	-	ALA144, VAL17, VAL18, VAL64, ASN132, LEU134, GLY11, GLY13, GLY16, ILE10, ILE35, LYS33, LYS89, GLU12, GLU162, GLN131, TYR15, THR14, ASP145, PHE80
CDK2 (1HCK)	Roscovitine	−131.669	−7.825	GLY153, GLY16, VAL18, LYS33, TYR15, ASP145, ILE10, GLY11, GLU12, LYS129, GLN131, GLU162, THR165, THR14, GLY13, TYR159, ASP127, ILE35, PHE152
Cyclin E (1W98)	8a	−146.952	-	GLN131, ASN132, VAL18, VAL64, LYS33, ALA31, ALA144, ASP145, PHE82, PHE146, PHE80,, GLU12, GLU81, LEU55, LEU83, LEU134, GLN85, ASP86, ILE10, GLY11, GLY13
Cyclin E (1W98)	Roscovitine	−147.197	−11.528	GLU81, VAL64, PHE80, ALA31, LYS33, VAL18, ALA144, HIS84, ILE10, GLN85, ASP86, LEU131, ASP86, ASP145, GLY13, TYR15, THR14, LYS129, ASN132, GLN131, GLU12, GLY11, PHE82, LEU83

## Discussion

This study investigated the synthesis and anticancer activity of novel benzimidazole derivatives in human breast cancer cell lines. Among the synthesized derivatives, compound 8a exhibited the most promising results, significantly inhibiting cell proliferation in both MCF-7 and MDA-MB-231 cell lines. Compound 8a showed a preferential antiproliferative effect toward cancer cells compared to the non-tumorigenic MCF-10A cell line, supporting its potential as a targeted anticancer candidate. Although compound 8a demonstrated moderate selectivity in vitro, such profiles are frequently observed at the early stages of anticancer drug development and may be further improved through structural optimization and evaluation in advanced biological models.

The molecular design of the investigated compounds was guided by the dual objective of simultaneously targeting cell cycle progression and apoptotic resistance, two interconnected hallmarks of breast cancer chemoresistance. The benzimidazole core was selected as the central scaffold due to its privileged heterocyclic structure, which is well known to engage ATP-binding pockets of cyclin-dependent kinases as well as hydrophobic grooves of apoptosis-regulating proteins. Structurally, the planar aromatic nature of the benzimidazole ring facilitates π–π stacking and hydrogen bonding interactions within kinase active sites, while appropriate substitution at the N-1 and C-2 positions enables extension toward the BH3-binding groove of anti-apoptotic Bcl-2 family proteins. Unlike previously reported benzimidazole derivatives that predominantly focus on a single biological pathway, the present design strategy intentionally integrates structural features capable of engaging both CDK-mediated cell cycle regulation and Bcl-2-mediated apoptotic resistance. This rational design hypothesis was further evaluated through combined in vitro mechanistic assays and molecular docking studies, allowing a direct correlation between structural features and dual biological activity.

Our observations corroborate previous research that has highlighted the anticancer activity of benzimidazole-based compounds. For instance, Atmaca et al. observed that specific benzimidazole derivatives are capable of inducing apoptosis and cell cycle arrest across a range of cancer cell lines, with these effects primarily mediated through the activation of the mitochondrial pathway and the subsequent generation of reactive oxygen species (ROS) (Atmaca et al. 2020). In a similar vein, Wang et al. demonstrated that benzimidazole scaffolds exhibit a strong binding affinity to cyclin-dependent kinases (CDKs), leading to superior anti-tumor activity in breast cancer cell lines (Wang et al. 2024).

In this study, treatment with compound 8a resulted in G2/M phase accumulation in both investigated breast cancer cell lines, indicating its role in cell cycle regulation. This suggests that 8a interferes with mitotic progression, likely through the inhibition of CDK2 and Cyclin E. Our molecular docking analysis supports this mechanism, revealing strong binding interactions between 8a and these key regulatory proteins. The role of CDK2 and Cyclin E in the G1/S transition is well-documented, and their inhibition is known to promote G2/M arrest and apoptosis in rapidly dividing cells, further reinforcing the proposed mode of action for compound 8a (Peyressatre et al. 2015; Hagar et al. 2024).

Furthermore, compound 8a significantly increased the percentage of apoptotic cells, indicating that its anticancer activity also involves the induction of programmed cell death. Flow cytometry results showed an increase in both early and late apoptotic populations. Consistent with our observations, benzimidazole derivatives have previously been reported to modulate apoptosis-related proteins such as Bcl-2 and Bcl-xL, shifting the balance towards cell death (Hasanpourghadi et al. 2016; Li et al. 2011; Nagy et al. 2021; Abbade et al. 2024).

The molecular docking analysis provided insights into potential molecular targets. Derivative 8a exhibited high binding affinity to Cyclin E and CDK2, reinforcing its role in cell cycle modulation. Moreover, interaction with anti-apoptotic proteins such as Bcl-2 suggests that this compound may inhibit survival pathways. This dual mechanism-cell cycle arrest and apoptosis induction-enhances its therapeutic relevance. Previous studies on dual-targeting benzimidazole derivatives have shown similar mechanisms of action, validating our approach (Alzahrani et al. 2022; Nazreen et al. 2022).

Overall, these results suggest that compound 8a holds promise as a candidate for further drug development. Nonetheless, additional in vivo investigations and structure–activity relationship (SAR) studies are essential to confirm its therapeutic efficacy and enhance its pharmacokinetic characteristics.

Despite the promising biological and computational findings, several limitations and challenges of the present study should be acknowledged. First, the anticancer activity of compound 8a was evaluated exclusively under in vitro conditions, which limits conclusions regarding its in vivo efficacy, pharmacokinetics, bioavailability, and systemic toxicity. Second, while molecular docking analyses provide valuable insight into potential target interactions, these results remain predictive in nature and require experimental validation through biochemical assays and pathway-level studies. In addition, the concentration ranges used in the cellular assays, although guided by IC₅₀ values and preliminary screening, may not fully reflect physiological exposure conditions. Finally, although compound 8a demonstrated selectivity toward cancer cells, a broader evaluation across additional normal cell models would further strengthen its safety profile. Addressing these challenges through in vivo studies and expanded structure–activity relationship analyses will be essential for advancing this compound toward translational development.

The present study was designed as a proof-of-concept investigation to identify and mechanistically characterize promising benzimidazole-based anticancer candidates at the cellular level. While in vivo models provide critical information regarding pharmacokinetics, bioavailability, and systemic toxicity, such studies require prior identification of lead compounds with reproducible in vitro efficacy and selectivity. In this context, compound 8a emerged as a lead candidate based on its dual effects on cell cycle arrest and apoptosis induction. Comprehensive in vivo evaluation will therefore be the focus of future studies to further validate its therapeutic potential.

## Conclusion

In summary, the findings of this study highlight the anticancer potential of the newly developed benzimidazole derivatives, with compound 8a emerging as a promising agent due to its significant cytotoxic effect on breast cancer cells and minimal adverse effects on healthy cells. Compound 8a demonstrates a dual-targeting mechanism, as evidenced by its capacity to induce G2/M phase arrest and promote apoptosis and its strong binding interactions with Cyclin E, CDK2, Bcl-2, and Bcl-xL. These findings identify compound 8a as a promising lead scaffold for the development of targeted breast cancer therapies. Further in vivo validation and structure–activity relationship studies are warranted to enhance its pharmacological profile and therapeutic applicability.

## Supplementary Information

Below is the link to the electronic supplementary material.

Supplementary file1 (TIF 1263 KB)

Supplementary file2 (DOCX 1328 KB)

Supplementary file3 (TIF 112 KB)

## Data Availability

The data generated for the current study are available from the corresponding author on reasonable request.
