# Supplementary figures and images for: Dual inhibition of cell cycle progression and apoptotic resistance in breast cancer by novel benzimidazole-based therapeutics
Source: Naunyn Schmiedebergs Arch Pharmacol. 2026 Mar 18;399(8):12673–85. doi: 10.1007/s00210-026-05206-y (PMC13269478; doi:10.1007/s00210-026-05206-y)

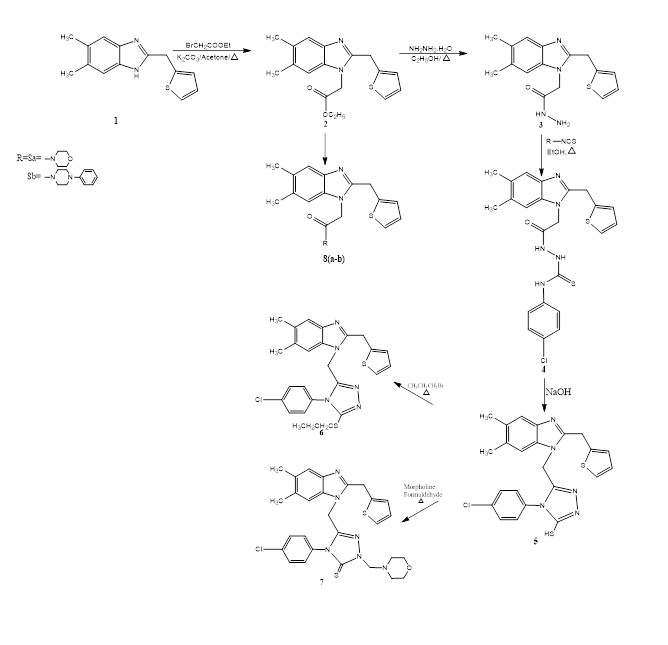

Supplement: Supplementary file 1 — Supplementary file1 (TIF 1263 KB) [file 210_2026_5206_MOESM1_ESM.tif]

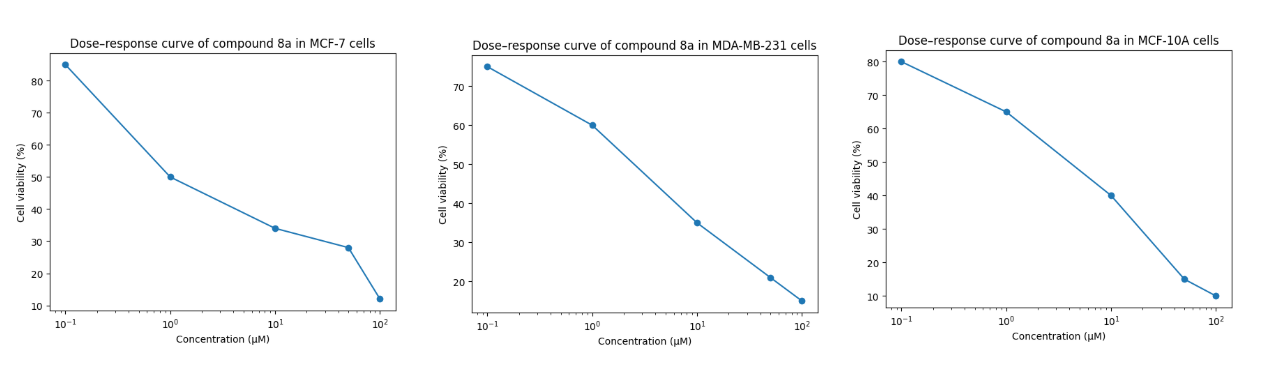

Supplement: Supplementary file 3 — Supplementary file3 (TIF 112 KB) [file 210_2026_5206_MOESM3_ESM.tif]
