# Supplementary material for: Dual inhibition of cell cycle progression and apoptotic resistance in breast cancer by novel benzimidazole-based therapeutics
Source: Naunyn Schmiedebergs Arch Pharmacol. 2026 Mar 18;399(8):12673–85. doi: 10.1007/s00210-026-05206-y (PMC13269478; doi:10.1007/s00210-026-05206-y)
Supplement: Supplementary file 2 — Supplementary file2 (DOCX 1328 KB) [file 210_2026_5206_MOESM2_ESM.docx]

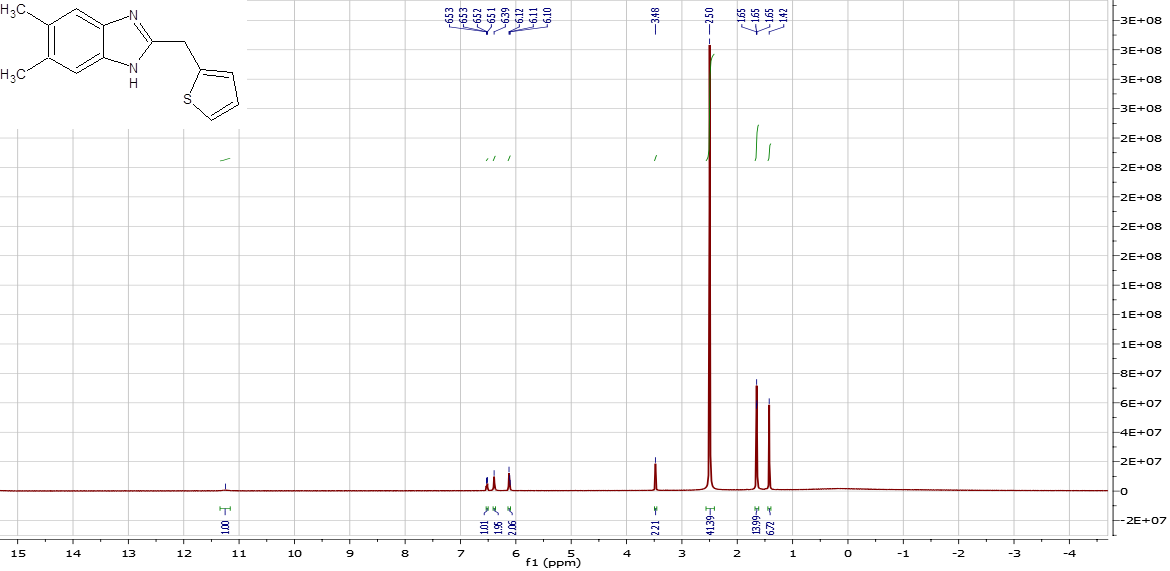


Compound 1 ^1^H NMR Spectrum, 400 MHz, DMSO-d6


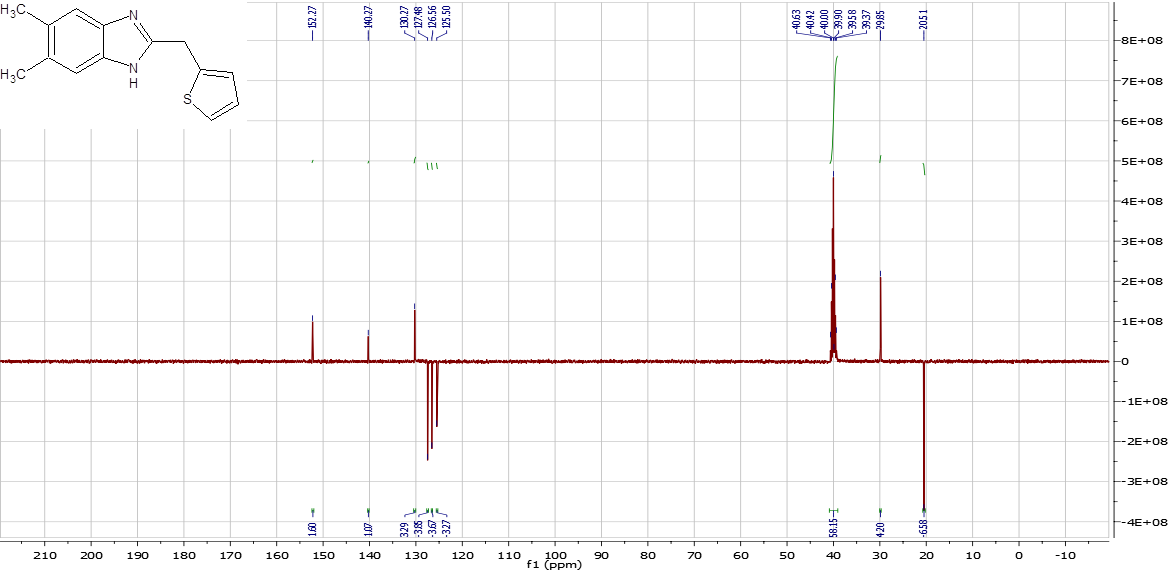


Compound 1 ^13^C APT NMR Spectrum, 100 MHz, DMSO-d6

1 Nolu Bileşiğin ^1^H NMR Spektrumu, 100 MHz, DMSO-d6

1 Nolu Bileşiğin ^1^H NMR Spektrumu, 100 MHz, DMSO-d6

1 Nolu Bileşiğin ^1^H NMR Spektrumu, 100 MHz, DMSO-d6

1 Nolu Bileşiğin ^1^H NMR Spektrumu, 100 MHz, DMSO-d6

1 Nolu Bileşiğin ^1^H NMR Spektrumu, 100 MHz, DMSO-d6

1 Nolu Bileşiğin ^1^H NMR Spektrumu, 100 MHz, DMSO-d6

1 Nolu Bileşiğin ^1^H NMR Spektrumu, 100 MHz, DMSO-d6

1 Nolu Bileşiğin ^1^H NMR Spektrumu, 100 MHz, DMSO-d6

1 Nolu Bileşiğin ^1^H NMR Spektrumu, 100 MHz, DMSO-d6

1 Nolu Bileşiğin ^1^H NMR Spektrumu, 100 MHz, DMSO-d6

1 Nolu Bileşiğin ^1^H NMR Spektrumu, 100 MHz, DMSO-d6

1 Nolu Bileşiğin ^1^H NMR Spektrumu, 100 MHz, DMSO-d6

1 Nolu Bileşiğin ^1^H NMR Spektrumu, 100 MHz, DMSO-d6

1 Nolu Bileşiğin ^1^H NMR Spektrumu, 100 MHz, DMSO-d6

1 Nolu Bileşiğin ^1^H NMR Spektrumu, 100 MHz, DMSO-d6

1 Nolu Bileşiğin ^1^H NMR Spektrumu, 100 MHz, DMSO-d6

1 Nolu Bileşiğin ^1^H NMR Spektrumu, 100 MHz, DMSO-d6

1 Nolu Bileşiğin ^1^H NMR Spektrumu, 100 MHz, DMSO-d6

1 Nolu Bileşiğin ^1^H NMR Spektrumu, 100 MHz, DMSO-d6

1 Nolu Bileşiğin ^1^H NMR Spektrumu, 100 MHz, DMSO-d6

1 Nolu Bileşiğin ^1^H NMR Spektrumu, 100 MHz, DMSO-d6

1 Nolu Bileşiğin ^1^H NMR Spektrumu, 100 MHz, DMSO-d6

1 Nolu Bileşiğin ^1^H NMR Spektrumu, 100 MHz, DMSO-d6

1 Nolu Bileşiğin ^1^H NMR Spektrumu, 100 MHz, DMSO-d6

1 Nolu Bileşiğin ^1^H NMR Spektrumu, 100 MHz, DMSO-d6

1 Nolu Bileşiğin ^1^H NMR Spektrumu, 100 MHz, DMSO-d6

1 Nolu Bileşiğin ^1^H NMR Spektrumu, 100 MHz, DMSO-d6

1 Nolu Bileşiğin ^1^H NMR Spektrumu, 100 MHz, DMSO-d6

1 Nolu Bileşiğin ^1^H NMR Spektrumu, 100 MHz, DMSO-d6

1 Nolu Bileşiğin ^1^H NMR Spektrumu, 100 MHz, DMSO-d6

1 Nolu Bileşiğin ^1^H NMR Spektrumu, 100 MHz, DMSO-d6


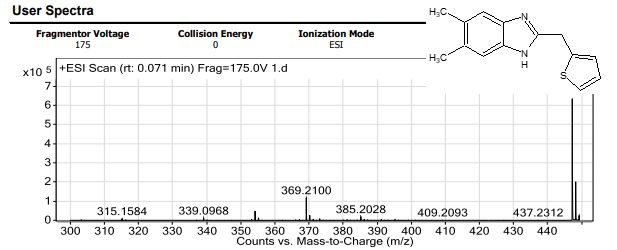


Compound 1 LC/TOF-MS

1 Nolu Bileşiğin Kütle Spektrumu

1 Nolu Bileşiğin Kütle Spektrumu

1 Nolu Bileşiğin Kütle Spektrumu

1 Nolu Bileşiğin Kütle Spektrumu

1 Nolu Bileşiğin Kütle Spektrumu

1 Nolu Bileşiğin Kütle Spektrumu

1 Nolu Bileşiğin Kütle Spektrumu

1 Nolu Bileşiğin Kütle Spektrumu

1 Nolu Bileşiğin Kütle Spektrumu

1 Nolu Bileşiğin Kütle Spektrumu

1 Nolu Bileşiğin Kütle Spektrumu

1 Nolu Bileşiğin Kütle Spektrumu

1 Nolu Bileşiğin Kütle Spektrumu

1 Nolu Bileşiğin Kütle Spektrumu

1 Nolu Bileşiğin Kütle Spektrumu

1 Nolu Bileşiğin Kütle Spektrumu

1 Nolu Bileşiğin Kütle Spektrumu

1 Nolu Bileşiğin Kütle Spektrumu

1 Nolu Bileşiğin Kütle Spektrumu

1 Nolu Bileşiğin Kütle Spektrumu

1 Nolu Bileşiğin Kütle Spektrumu

1 Nolu Bileşiğin Kütle Spektrumu

1 Nolu Bileşiğin Kütle Spektrumu

1 Nolu Bileşiğin Kütle Spektrumu

1 Nolu Bileşiğin Kütle Spektrumu

1 Nolu Bileşiğin Kütle Spektrumu

1 Nolu Bileşiğin Kütle Spektrumu

1 Nolu Bileşiğin Kütle Spektrumu

1 Nolu Bileşiğin Kütle Spektrumu

1 Nolu Bileşiğin Kütle Spektrumu

1 Nolu Bileşiğin Kütle Spektrumu


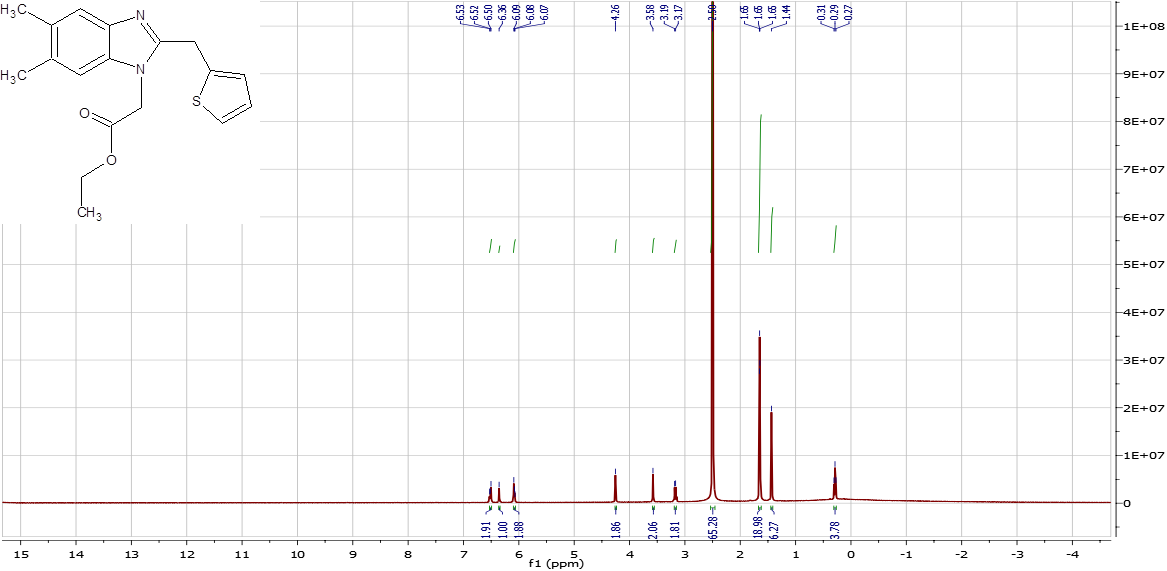


Compound 2 ^1^H NMR Spectrum, 400 MHz, DMSO-d6


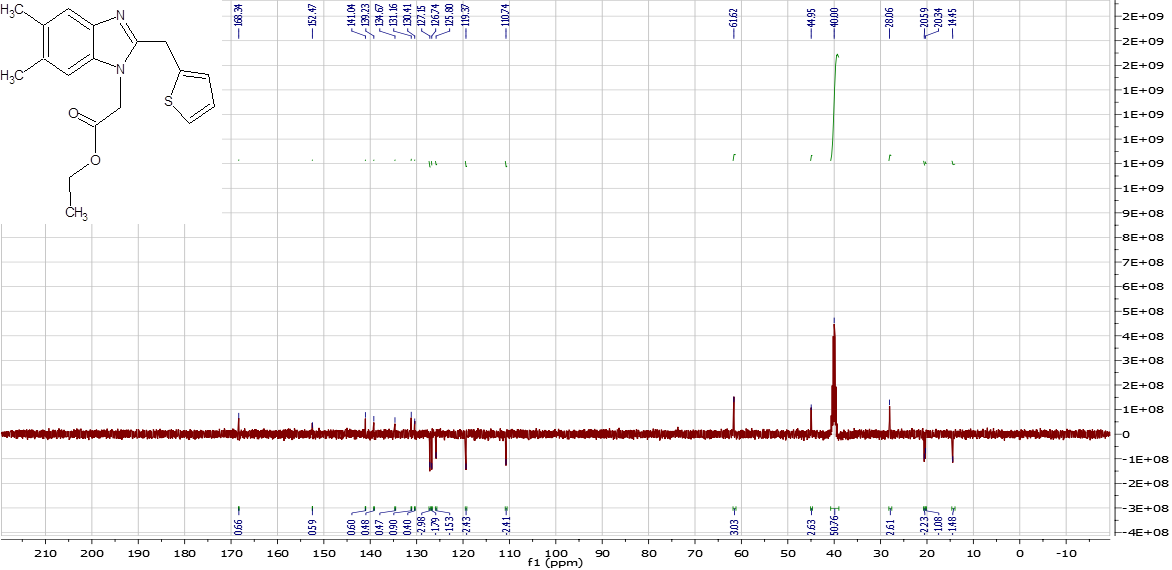


Compound 2 ^13^C APT NMR Spectrum, 100 MHz, DMSO-d6

1 Nolu Bileşiğin ^1^H NMR Spektrumu, 100 MHz, DMSO-d6

1 Nolu Bileşiğin ^1^H NMR Spektrumu, 100 MHz, DMSO-d6

1 Nolu Bileşiğin ^1^H NMR Spektrumu, 100 MHz, DMSO-d6

1 Nolu Bileşiğin ^1^H NMR Spektrumu, 100 MHz, DMSO-d6

1 Nolu Bileşiğin ^1^H NMR Spektrumu, 100 MHz, DMSO-d6

1 Nolu Bileşiğin ^1^H NMR Spektrumu, 100 MHz, DMSO-d6

1 Nolu Bileşiğin ^1^H NMR Spektrumu, 100 MHz, DMSO-d6

1 Nolu Bileşiğin ^1^H NMR Spektrumu, 100 MHz, DMSO-d6

1 Nolu Bileşiğin ^1^H NMR Spektrumu, 100 MHz, DMSO-d6

1 Nolu Bileşiğin ^1^H NMR Spektrumu, 100 MHz, DMSO-d6

1 Nolu Bileşiğin ^1^H NMR Spektrumu, 100 MHz, DMSO-d6

1 Nolu Bileşiğin ^1^H NMR Spektrumu, 100 MHz, DMSO-d6

1 Nolu Bileşiğin ^1^H NMR Spektrumu, 100 MHz, DMSO-d6

1 Nolu Bileşiğin ^1^H NMR Spektrumu, 100 MHz, DMSO-d6

1 Nolu Bileşiğin ^1^H NMR Spektrumu, 100 MHz, DMSO-d6

1 Nolu Bileşiğin ^1^H NMR Spektrumu, 100 MHz, DMSO-d6

1 Nolu Bileşiğin ^1^H NMR Spektrumu, 100 MHz, DMSO-d6

1 Nolu Bileşiğin ^1^H NMR Spektrumu, 100 MHz, DMSO-d6

1 Nolu Bileşiğin ^1^H NMR Spektrumu, 100 MHz, DMSO-d6

1 Nolu Bileşiğin ^1^H NMR Spektrumu, 100 MHz, DMSO-d6

1 Nolu Bileşiğin ^1^H NMR Spektrumu, 100 MHz, DMSO-d6

1 Nolu Bileşiğin ^1^H NMR Spektrumu, 100 MHz, DMSO-d6

1 Nolu Bileşiğin ^1^H NMR Spektrumu, 100 MHz, DMSO-d6

1 Nolu Bileşiğin ^1^H NMR Spektrumu, 100 MHz, DMSO-d6

1 Nolu Bileşiğin ^1^H NMR Spektrumu, 100 MHz, DMSO-d6

1 Nolu Bileşiğin ^1^H NMR Spektrumu, 100 MHz, DMSO-d6

1 Nolu Bileşiğin ^1^H NMR Spektrumu, 100 MHz, DMSO-d6

1 Nolu Bileşiğin ^1^H NMR Spektrumu, 100 MHz, DMSO-d6

1 Nolu Bileşiğin ^1^H NMR Spektrumu, 100 MHz, DMSO-d6

1 Nolu Bileşiğin ^1^H NMR Spektrumu, 100 MHz, DMSO-d6

1 Nolu Bileşiğin ^1^H NMR Spektrumu, 100 MHz, DMSO-d6


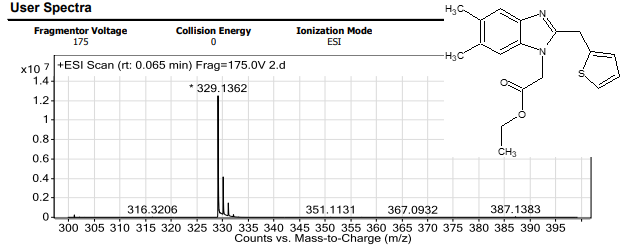


Compound 2 LC/TOF-MS

1 Nolu Bileşiğin Kütle Spektrumu

1 Nolu Bileşiğin Kütle Spektrumu

1 Nolu Bileşiğin Kütle Spektrumu

1 Nolu Bileşiğin Kütle Spektrumu

1 Nolu Bileşiğin Kütle Spektrumu

1 Nolu Bileşiğin Kütle Spektrumu

1 Nolu Bileşiğin Kütle Spektrumu

1 Nolu Bileşiğin Kütle Spektrumu

1 Nolu Bileşiğin Kütle Spektrumu

1 Nolu Bileşiğin Kütle Spektrumu

1 Nolu Bileşiğin Kütle Spektrumu

1 Nolu Bileşiğin Kütle Spektrumu

1 Nolu Bileşiğin Kütle Spektrumu

1 Nolu Bileşiğin Kütle Spektrumu

1 Nolu Bileşiğin Kütle Spektrumu

1 Nolu Bileşiğin Kütle Spektrumu

1 Nolu Bileşiğin Kütle Spektrumu

1 Nolu Bileşiğin Kütle Spektrumu

1 Nolu Bileşiğin Kütle Spektrumu

1 Nolu Bileşiğin Kütle Spektrumu

1 Nolu Bileşiğin Kütle Spektrumu

1 Nolu Bileşiğin Kütle Spektrumu

1 Nolu Bileşiğin Kütle Spektrumu

1 Nolu Bileşiğin Kütle Spektrumu

1 Nolu Bileşiğin Kütle Spektrumu

1 Nolu Bileşiğin Kütle Spektrumu

1 Nolu Bileşiğin Kütle Spektrumu

1 Nolu Bileşiğin Kütle Spektrumu

1 Nolu Bileşiğin Kütle Spektrumu

1 Nolu Bileşiğin Kütle Spektrumu

1 Nolu Bileşiğin Kütle Spektrumu


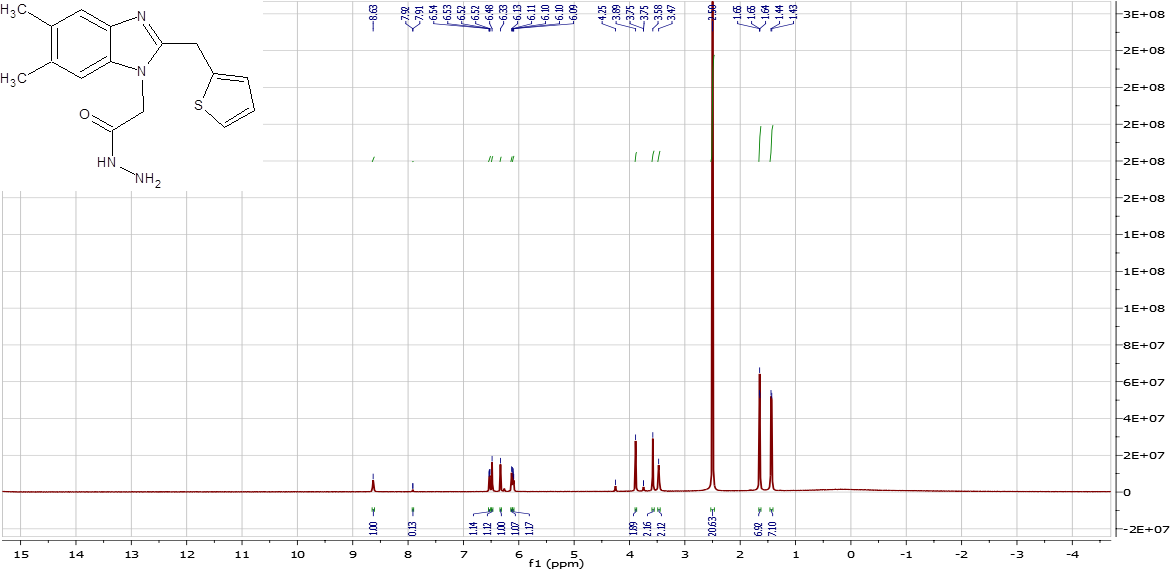


Compound 3 ^1^H NMR Spectrum, 400 MHz, DMSO-d6


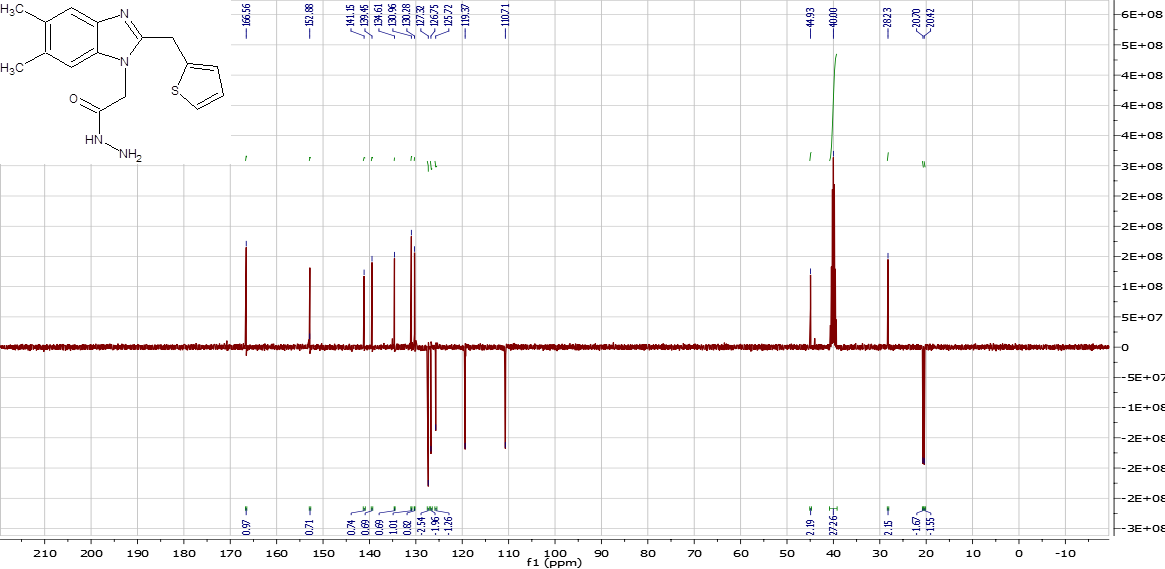


Compound 3 ^13^C APT NMR Spectrum, 100 MHz, DMSO-d6

1 Nolu Bileşiğin ^1^H NMR Spektrumu, 100 MHz, DMSO-d6

1 Nolu Bileşiğin ^1^H NMR Spektrumu, 100 MHz, DMSO-d6

1 Nolu Bileşiğin ^1^H NMR Spektrumu, 100 MHz, DMSO-d6

1 Nolu Bileşiğin ^1^H NMR Spektrumu, 100 MHz, DMSO-d6

1 Nolu Bileşiğin ^1^H NMR Spektrumu, 100 MHz, DMSO-d6

1 Nolu Bileşiğin ^1^H NMR Spektrumu, 100 MHz, DMSO-d6

1 Nolu Bileşiğin ^1^H NMR Spektrumu, 100 MHz, DMSO-d6

1 Nolu Bileşiğin ^1^H NMR Spektrumu, 100 MHz, DMSO-d6

1 Nolu Bileşiğin ^1^H NMR Spektrumu, 100 MHz, DMSO-d6

1 Nolu Bileşiğin ^1^H NMR Spektrumu, 100 MHz, DMSO-d6

1 Nolu Bileşiğin ^1^H NMR Spektrumu, 100 MHz, DMSO-d6

1 Nolu Bileşiğin ^1^H NMR Spektrumu, 100 MHz, DMSO-d6

1 Nolu Bileşiğin ^1^H NMR Spektrumu, 100 MHz, DMSO-d6

1 Nolu Bileşiğin ^1^H NMR Spektrumu, 100 MHz, DMSO-d6

1 Nolu Bileşiğin ^1^H NMR Spektrumu, 100 MHz, DMSO-d6

1 Nolu Bileşiğin ^1^H NMR Spektrumu, 100 MHz, DMSO-d6

1 Nolu Bileşiğin ^1^H NMR Spektrumu, 100 MHz, DMSO-d6

1 Nolu Bileşiğin ^1^H NMR Spektrumu, 100 MHz, DMSO-d6

1 Nolu Bileşiğin ^1^H NMR Spektrumu, 100 MHz, DMSO-d6

1 Nolu Bileşiğin ^1^H NMR Spektrumu, 100 MHz, DMSO-d6

1 Nolu Bileşiğin ^1^H NMR Spektrumu, 100 MHz, DMSO-d6

1 Nolu Bileşiğin ^1^H NMR Spektrumu, 100 MHz, DMSO-d6

1 Nolu Bileşiğin ^1^H NMR Spektrumu, 100 MHz, DMSO-d6

1 Nolu Bileşiğin ^1^H NMR Spektrumu, 100 MHz, DMSO-d6

1 Nolu Bileşiğin ^1^H NMR Spektrumu, 100 MHz, DMSO-d6

1 Nolu Bileşiğin ^1^H NMR Spektrumu, 100 MHz, DMSO-d6

1 Nolu Bileşiğin ^1^H NMR Spektrumu, 100 MHz, DMSO-d6

1 Nolu Bileşiğin ^1^H NMR Spektrumu, 100 MHz, DMSO-d6

1 Nolu Bileşiğin ^1^H NMR Spektrumu, 100 MHz, DMSO-d6

1 Nolu Bileşiğin ^1^H NMR Spektrumu, 100 MHz, DMSO-d6

1 Nolu Bileşiğin ^1^H NMR Spektrumu, 100 MHz, DMSO-d6


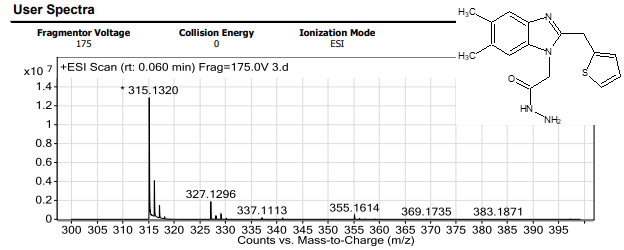


Compound 3 LC/TOF-MS

1 Nolu Bileşiğin Kütle Spektrumu

1 Nolu Bileşiğin Kütle Spektrumu

1 Nolu Bileşiğin Kütle Spektrumu

1 Nolu Bileşiğin Kütle Spektrumu

1 Nolu Bileşiğin Kütle Spektrumu

1 Nolu Bileşiğin Kütle Spektrumu

1 Nolu Bileşiğin Kütle Spektrumu

1 Nolu Bileşiğin Kütle Spektrumu

1 Nolu Bileşiğin Kütle Spektrumu

1 Nolu Bileşiğin Kütle Spektrumu

1 Nolu Bileşiğin Kütle Spektrumu

1 Nolu Bileşiğin Kütle Spektrumu

1 Nolu Bileşiğin Kütle Spektrumu

1 Nolu Bileşiğin Kütle Spektrumu

1 Nolu Bileşiğin Kütle Spektrumu

1 Nolu Bileşiğin Kütle Spektrumu

1 Nolu Bileşiğin Kütle Spektrumu

1 Nolu Bileşiğin Kütle Spektrumu

1 Nolu Bileşiğin Kütle Spektrumu

1 Nolu Bileşiğin Kütle Spektrumu

1 Nolu Bileşiğin Kütle Spektrumu

1 Nolu Bileşiğin Kütle Spektrumu

1 Nolu Bileşiğin Kütle Spektrumu

1 Nolu Bileşiğin Kütle Spektrumu

1 Nolu Bileşiğin Kütle Spektrumu

1 Nolu Bileşiğin Kütle Spektrumu

1 Nolu Bileşiğin Kütle Spektrumu

1 Nolu Bileşiğin Kütle Spektrumu

1 Nolu Bileşiğin Kütle Spektrumu

1 Nolu Bileşiğin Kütle Spektrumu

1 Nolu Bileşiğin Kütle Spektrumu


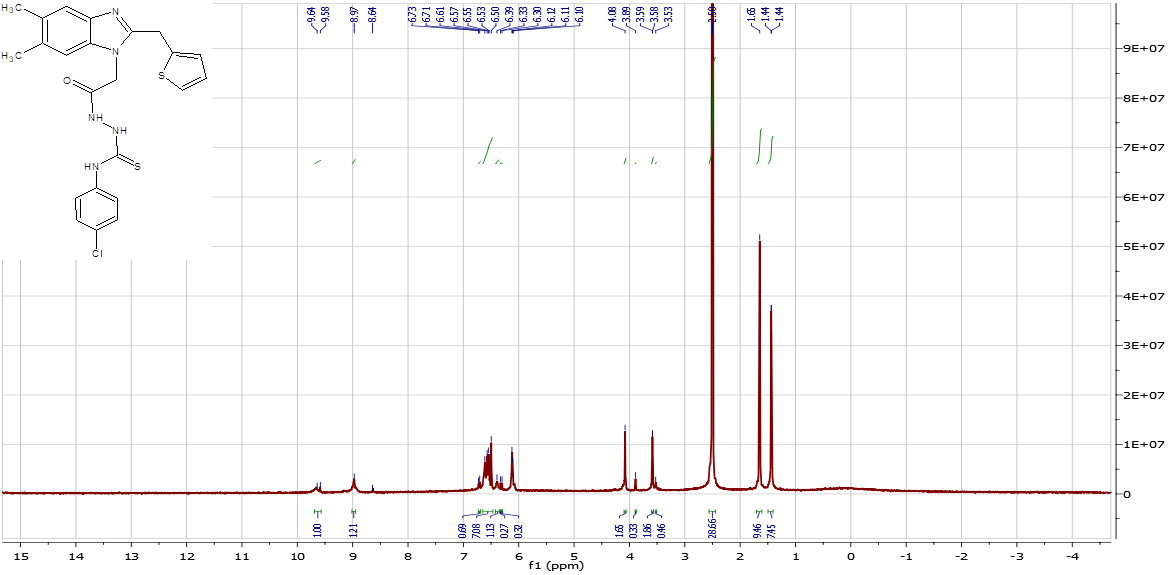


Compound 4 ^1^H NMR Spectrum, 400 MHz, DMSO-d6


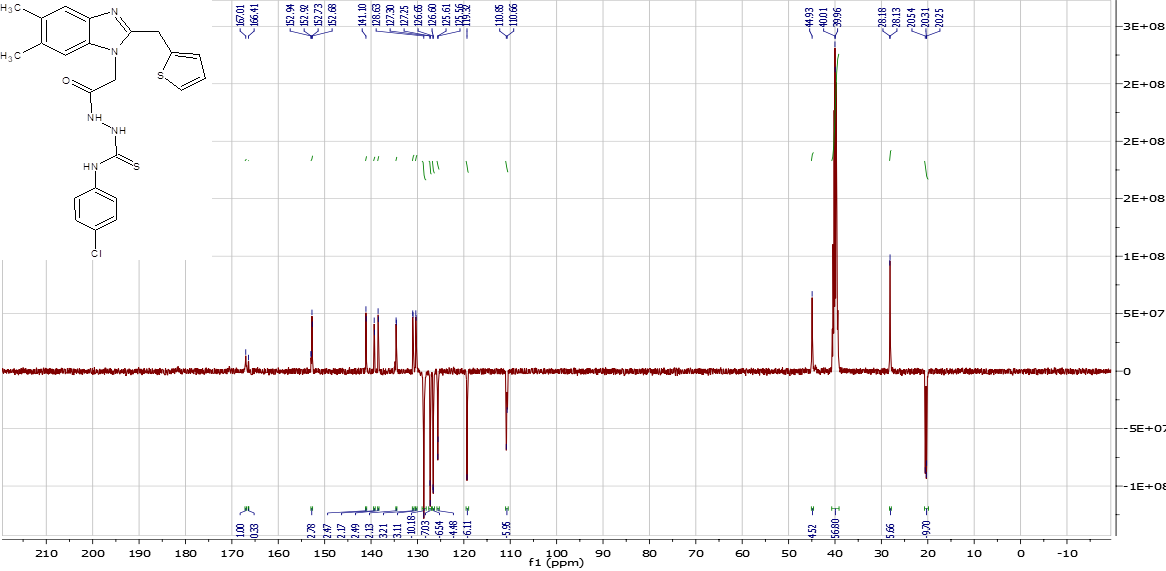


Compound 4 ^13^C APT NMR Spectrum, 100 MHz, DMSO-d6

1 Nolu Bileşiğin ^1^H NMR Spektrumu, 100 MHz, DMSO-d6

1 Nolu Bileşiğin ^1^H NMR Spektrumu, 100 MHz, DMSO-d6

1 Nolu Bileşiğin ^1^H NMR Spektrumu, 100 MHz, DMSO-d6

1 Nolu Bileşiğin ^1^H NMR Spektrumu, 100 MHz, DMSO-d6

1 Nolu Bileşiğin ^1^H NMR Spektrumu, 100 MHz, DMSO-d6

1 Nolu Bileşiğin ^1^H NMR Spektrumu, 100 MHz, DMSO-d6

1 Nolu Bileşiğin ^1^H NMR Spektrumu, 100 MHz, DMSO-d6

1 Nolu Bileşiğin ^1^H NMR Spektrumu, 100 MHz, DMSO-d6

1 Nolu Bileşiğin ^1^H NMR Spektrumu, 100 MHz, DMSO-d6

1 Nolu Bileşiğin ^1^H NMR Spektrumu, 100 MHz, DMSO-d6

1 Nolu Bileşiğin ^1^H NMR Spektrumu, 100 MHz, DMSO-d6

1 Nolu Bileşiğin ^1^H NMR Spektrumu, 100 MHz, DMSO-d6

1 Nolu Bileşiğin ^1^H NMR Spektrumu, 100 MHz, DMSO-d6

1 Nolu Bileşiğin ^1^H NMR Spektrumu, 100 MHz, DMSO-d6

1 Nolu Bileşiğin ^1^H NMR Spektrumu, 100 MHz, DMSO-d6

1 Nolu Bileşiğin ^1^H NMR Spektrumu, 100 MHz, DMSO-d6

1 Nolu Bileşiğin ^1^H NMR Spektrumu, 100 MHz, DMSO-d6

1 Nolu Bileşiğin ^1^H NMR Spektrumu, 100 MHz, DMSO-d6

1 Nolu Bileşiğin ^1^H NMR Spektrumu, 100 MHz, DMSO-d6

1 Nolu Bileşiğin ^1^H NMR Spektrumu, 100 MHz, DMSO-d6

1 Nolu Bileşiğin ^1^H NMR Spektrumu, 100 MHz, DMSO-d6

1 Nolu Bileşiğin ^1^H NMR Spektrumu, 100 MHz, DMSO-d6

1 Nolu Bileşiğin ^1^H NMR Spektrumu, 100 MHz, DMSO-d6

1 Nolu Bileşiğin ^1^H NMR Spektrumu, 100 MHz, DMSO-d6

1 Nolu Bileşiğin ^1^H NMR Spektrumu, 100 MHz, DMSO-d6

1 Nolu Bileşiğin ^1^H NMR Spektrumu, 100 MHz, DMSO-d6

1 Nolu Bileşiğin ^1^H NMR Spektrumu, 100 MHz, DMSO-d6

1 Nolu Bileşiğin ^1^H NMR Spektrumu, 100 MHz, DMSO-d6

1 Nolu Bileşiğin ^1^H NMR Spektrumu, 100 MHz, DMSO-d6

1 Nolu Bileşiğin ^1^H NMR Spektrumu, 100 MHz, DMSO-d6

1 Nolu Bileşiğin ^1^H NMR Spektrumu, 100 MHz, DMSO-d6


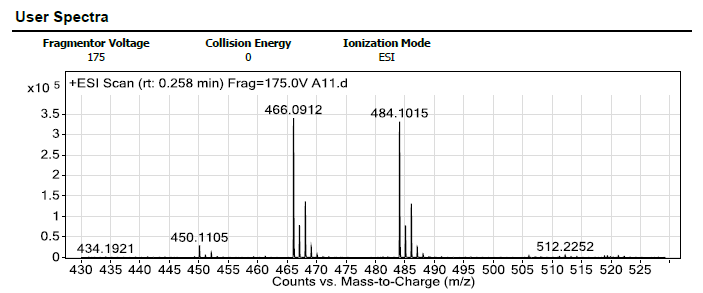


Compound 4 LC/TOF-MS

1 Nolu Bileşiğin Kütle Spektrumu

1 Nolu Bileşiğin Kütle Spektrumu

1 Nolu Bileşiğin Kütle Spektrumu

1 Nolu Bileşiğin Kütle Spektrumu

1 Nolu Bileşiğin Kütle Spektrumu

1 Nolu Bileşiğin Kütle Spektrumu

1 Nolu Bileşiğin Kütle Spektrumu

1 Nolu Bileşiğin Kütle Spektrumu

1 Nolu Bileşiğin Kütle Spektrumu

1 Nolu Bileşiğin Kütle Spektrumu

1 Nolu Bileşiğin Kütle Spektrumu

1 Nolu Bileşiğin Kütle Spektrumu

1 Nolu Bileşiğin Kütle Spektrumu

1 Nolu Bileşiğin Kütle Spektrumu

1 Nolu Bileşiğin Kütle Spektrumu

1 Nolu Bileşiğin Kütle Spektrumu

1 Nolu Bileşiğin Kütle Spektrumu

1 Nolu Bileşiğin Kütle Spektrumu

1 Nolu Bileşiğin Kütle Spektrumu

1 Nolu Bileşiğin Kütle Spektrumu

1 Nolu Bileşiğin Kütle Spektrumu

1 Nolu Bileşiğin Kütle Spektrumu

1 Nolu Bileşiğin Kütle Spektrumu

1 Nolu Bileşiğin Kütle Spektrumu

1 Nolu Bileşiğin Kütle Spektrumu

1 Nolu Bileşiğin Kütle Spektrumu

1 Nolu Bileşiğin Kütle Spektrumu

1 Nolu Bileşiğin Kütle Spektrumu

1 Nolu Bileşiğin Kütle Spektrumu

1 Nolu Bileşiğin Kütle Spektrumu

1 Nolu Bileşiğin Kütle Spektrumu


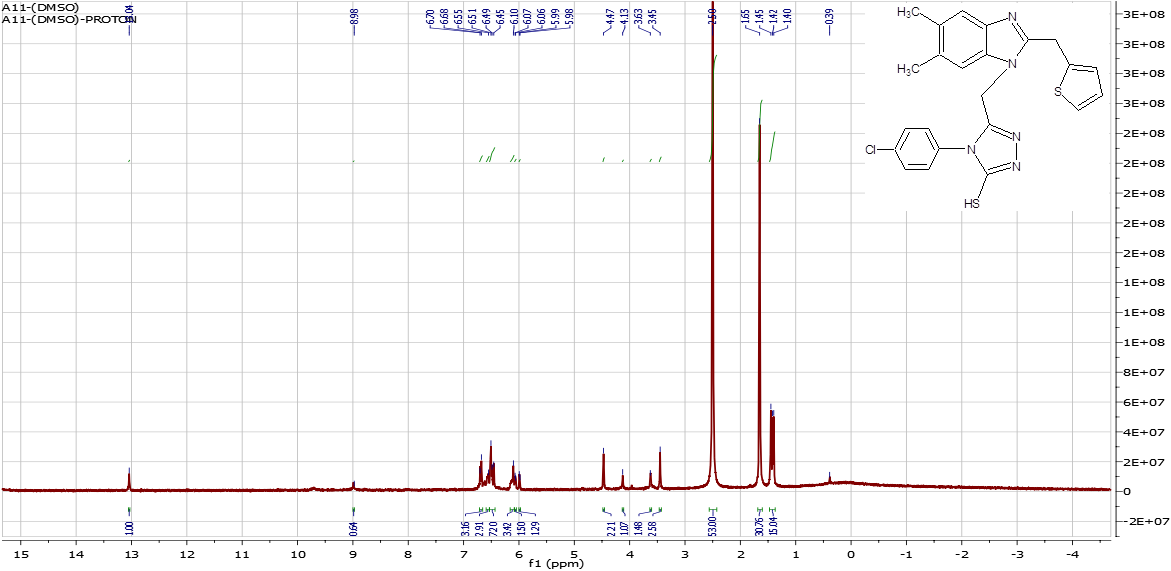


Compound 5 ^1^H NMR Spectrum, 400 MHz, DMSO-d6


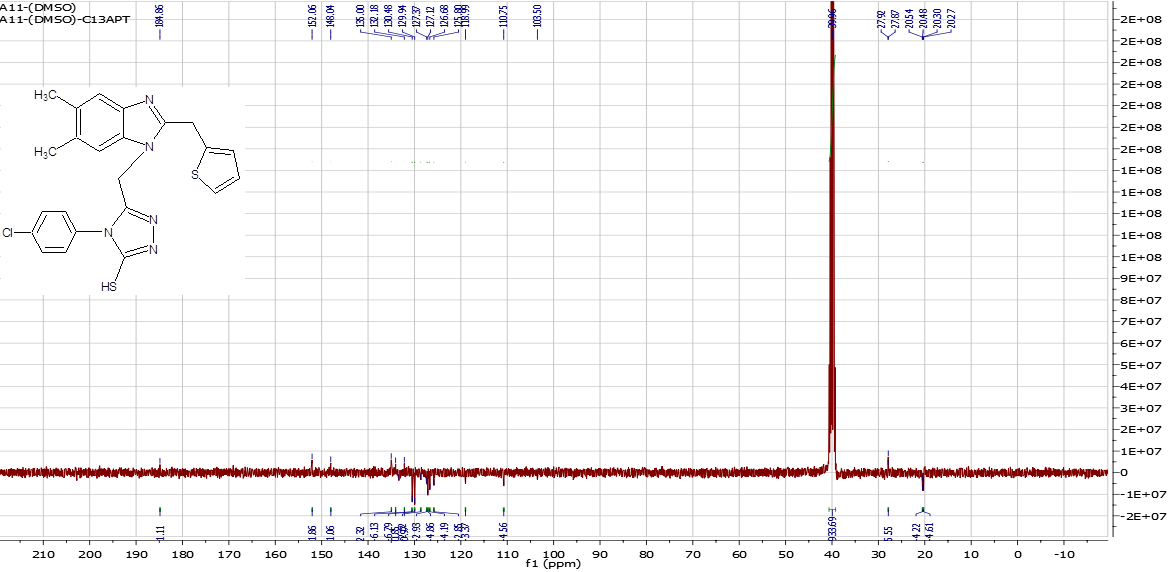


Compound 5 ^13^C APT NMR Spectrum, 100 MHz, DMSO-d6

1 Nolu Bileşiğin ^1^H NMR Spektrumu, 100 MHz, DMSO-d6

1 Nolu Bileşiğin ^1^H NMR Spektrumu, 100 MHz, DMSO-d6

1 Nolu Bileşiğin ^1^H NMR Spektrumu, 100 MHz, DMSO-d6

1 Nolu Bileşiğin ^1^H NMR Spektrumu, 100 MHz, DMSO-d6

1 Nolu Bileşiğin ^1^H NMR Spektrumu, 100 MHz, DMSO-d6

1 Nolu Bileşiğin ^1^H NMR Spektrumu, 100 MHz, DMSO-d6

1 Nolu Bileşiğin ^1^H NMR Spektrumu, 100 MHz, DMSO-d6

1 Nolu Bileşiğin ^1^H NMR Spektrumu, 100 MHz, DMSO-d6

1 Nolu Bileşiğin ^1^H NMR Spektrumu, 100 MHz, DMSO-d6

1 Nolu Bileşiğin ^1^H NMR Spektrumu, 100 MHz, DMSO-d6

1 Nolu Bileşiğin ^1^H NMR Spektrumu, 100 MHz, DMSO-d6

1 Nolu Bileşiğin ^1^H NMR Spektrumu, 100 MHz, DMSO-d6

1 Nolu Bileşiğin ^1^H NMR Spektrumu, 100 MHz, DMSO-d6

1 Nolu Bileşiğin ^1^H NMR Spektrumu, 100 MHz, DMSO-d6

1 Nolu Bileşiğin ^1^H NMR Spektrumu, 100 MHz, DMSO-d6

1 Nolu Bileşiğin ^1^H NMR Spektrumu, 100 MHz, DMSO-d6

1 Nolu Bileşiğin ^1^H NMR Spektrumu, 100 MHz, DMSO-d6

1 Nolu Bileşiğin ^1^H NMR Spektrumu, 100 MHz, DMSO-d6

1 Nolu Bileşiğin ^1^H NMR Spektrumu, 100 MHz, DMSO-d6

1 Nolu Bileşiğin ^1^H NMR Spektrumu, 100 MHz, DMSO-d6

1 Nolu Bileşiğin ^1^H NMR Spektrumu, 100 MHz, DMSO-d6

1 Nolu Bileşiğin ^1^H NMR Spektrumu, 100 MHz, DMSO-d6

1 Nolu Bileşiğin ^1^H NMR Spektrumu, 100 MHz, DMSO-d6

1 Nolu Bileşiğin ^1^H NMR Spektrumu, 100 MHz, DMSO-d6

1 Nolu Bileşiğin ^1^H NMR Spektrumu, 100 MHz, DMSO-d6

1 Nolu Bileşiğin ^1^H NMR Spektrumu, 100 MHz, DMSO-d6

1 Nolu Bileşiğin ^1^H NMR Spektrumu, 100 MHz, DMSO-d6

1 Nolu Bileşiğin ^1^H NMR Spektrumu, 100 MHz, DMSO-d6

1 Nolu Bileşiğin ^1^H NMR Spektrumu, 100 MHz, DMSO-d6

1 Nolu Bileşiğin ^1^H NMR Spektrumu, 100 MHz, DMSO-d6

1 Nolu Bileşiğin ^1^H NMR Spektrumu, 100 MHz, DMSO-d6


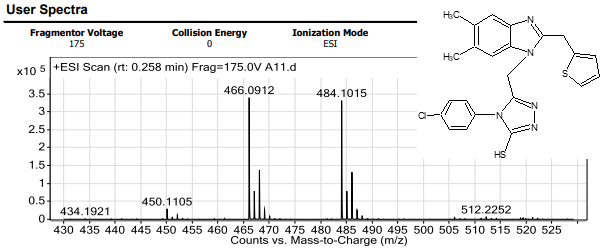


Compound 5 LC/TOF-MS

1 Nolu Bileşiğin Kütle Spektrumu

1 Nolu Bileşiğin Kütle Spektrumu

1 Nolu Bileşiğin Kütle Spektrumu

1 Nolu Bileşiğin Kütle Spektrumu

1 Nolu Bileşiğin Kütle Spektrumu

1 Nolu Bileşiğin Kütle Spektrumu

1 Nolu Bileşiğin Kütle Spektrumu

1 Nolu Bileşiğin Kütle Spektrumu

1 Nolu Bileşiğin Kütle Spektrumu

1 Nolu Bileşiğin Kütle Spektrumu

1 Nolu Bileşiğin Kütle Spektrumu

1 Nolu Bileşiğin Kütle Spektrumu

1 Nolu Bileşiğin Kütle Spektrumu

1 Nolu Bileşiğin Kütle Spektrumu

1 Nolu Bileşiğin Kütle Spektrumu

1 Nolu Bileşiğin Kütle Spektrumu

1 Nolu Bileşiğin Kütle Spektrumu

1 Nolu Bileşiğin Kütle Spektrumu

1 Nolu Bileşiğin Kütle Spektrumu

1 Nolu Bileşiğin Kütle Spektrumu

1 Nolu Bileşiğin Kütle Spektrumu

1 Nolu Bileşiğin Kütle Spektrumu

1 Nolu Bileşiğin Kütle Spektrumu

1 Nolu Bileşiğin Kütle Spektrumu

1 Nolu Bileşiğin Kütle Spektrumu

1 Nolu Bileşiğin Kütle Spektrumu

1 Nolu Bileşiğin Kütle Spektrumu

1 Nolu Bileşiğin Kütle Spektrumu

1 Nolu Bileşiğin Kütle Spektrumu

1 Nolu Bileşiğin Kütle Spektrumu

1 Nolu Bileşiğin Kütle Spektrumu


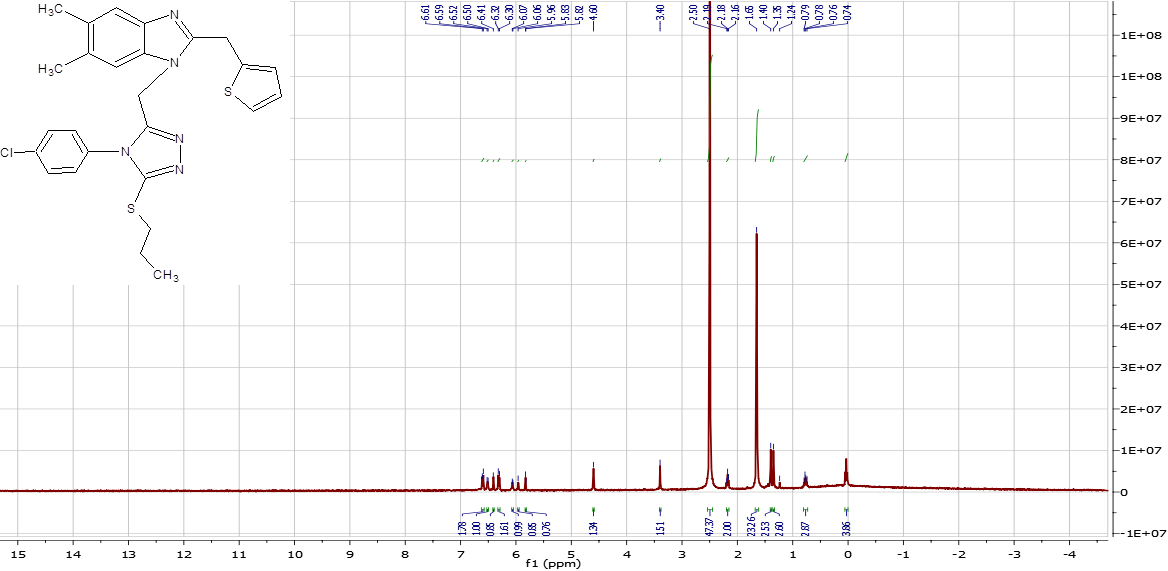


Compound 6 ^1^H NMR Spektrumu, 400 MHz, DMSO-d6


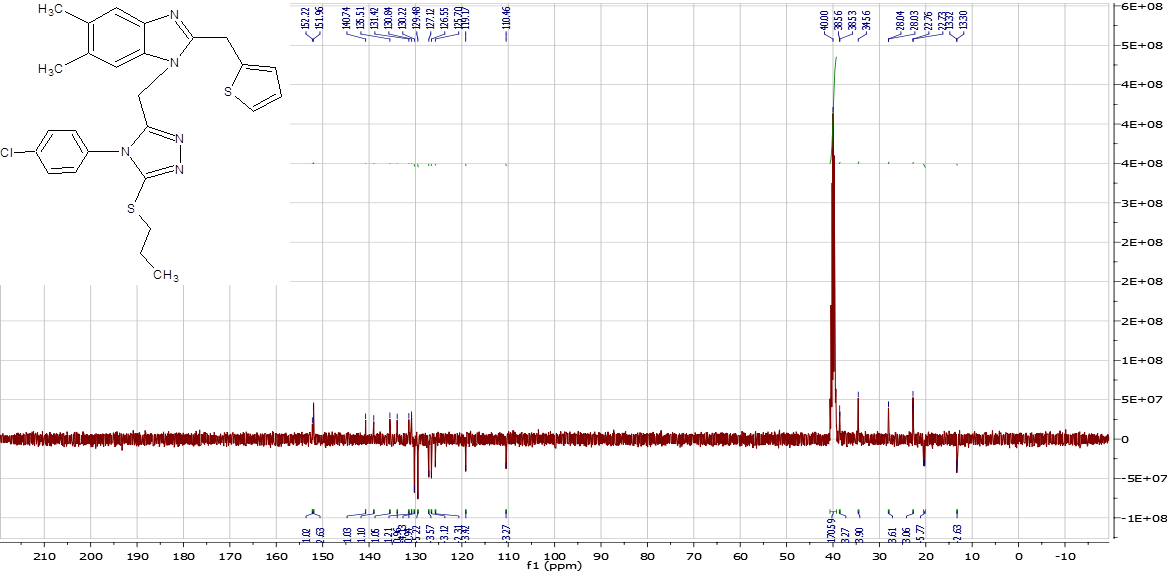


Compound 6 ^13^C APT NMR Spectrum, 100 MHz, DMSO-d6

1 Nolu Bileşiğin ^1^H NMR Spektrumu, 100 MHz, DMSO-d6

1 Nolu Bileşiğin ^1^H NMR Spektrumu, 100 MHz, DMSO-d6

1 Nolu Bileşiğin ^1^H NMR Spektrumu, 100 MHz, DMSO-d6

1 Nolu Bileşiğin ^1^H NMR Spektrumu, 100 MHz, DMSO-d6

1 Nolu Bileşiğin ^1^H NMR Spektrumu, 100 MHz, DMSO-d6

1 Nolu Bileşiğin ^1^H NMR Spektrumu, 100 MHz, DMSO-d6

1 Nolu Bileşiğin ^1^H NMR Spektrumu, 100 MHz, DMSO-d6

1 Nolu Bileşiğin ^1^H NMR Spektrumu, 100 MHz, DMSO-d6

1 Nolu Bileşiğin ^1^H NMR Spektrumu, 100 MHz, DMSO-d6

1 Nolu Bileşiğin ^1^H NMR Spektrumu, 100 MHz, DMSO-d6

1 Nolu Bileşiğin ^1^H NMR Spektrumu, 100 MHz, DMSO-d6

1 Nolu Bileşiğin ^1^H NMR Spektrumu, 100 MHz, DMSO-d6

1 Nolu Bileşiğin ^1^H NMR Spektrumu, 100 MHz, DMSO-d6

1 Nolu Bileşiğin ^1^H NMR Spektrumu, 100 MHz, DMSO-d6

1 Nolu Bileşiğin ^1^H NMR Spektrumu, 100 MHz, DMSO-d6

1 Nolu Bileşiğin ^1^H NMR Spektrumu, 100 MHz, DMSO-d6

1 Nolu Bileşiğin ^1^H NMR Spektrumu, 100 MHz, DMSO-d6

1 Nolu Bileşiğin ^1^H NMR Spektrumu, 100 MHz, DMSO-d6

1 Nolu Bileşiğin ^1^H NMR Spektrumu, 100 MHz, DMSO-d6

1 Nolu Bileşiğin ^1^H NMR Spektrumu, 100 MHz, DMSO-d6

1 Nolu Bileşiğin ^1^H NMR Spektrumu, 100 MHz, DMSO-d6

1 Nolu Bileşiğin ^1^H NMR Spektrumu, 100 MHz, DMSO-d6

1 Nolu Bileşiğin ^1^H NMR Spektrumu, 100 MHz, DMSO-d6

1 Nolu Bileşiğin ^1^H NMR Spektrumu, 100 MHz, DMSO-d6

1 Nolu Bileşiğin ^1^H NMR Spektrumu, 100 MHz, DMSO-d6

1 Nolu Bileşiğin ^1^H NMR Spektrumu, 100 MHz, DMSO-d6

1 Nolu Bileşiğin ^1^H NMR Spektrumu, 100 MHz, DMSO-d6

1 Nolu Bileşiğin ^1^H NMR Spektrumu, 100 MHz, DMSO-d6

1 Nolu Bileşiğin ^1^H NMR Spektrumu, 100 MHz, DMSO-d6

1 Nolu Bileşiğin ^1^H NMR Spektrumu, 100 MHz, DMSO-d6

1 Nolu Bileşiğin ^1^H NMR Spektrumu, 100 MHz, DMSO-d6


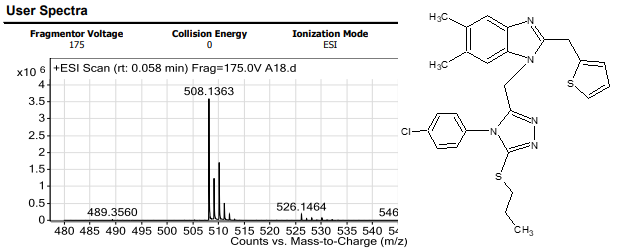


Compound 6 LC/TOF-MS

1 Nolu Bileşiğin Kütle Spektrumu

1 Nolu Bileşiğin Kütle Spektrumu

1 Nolu Bileşiğin Kütle Spektrumu

1 Nolu Bileşiğin Kütle Spektrumu

1 Nolu Bileşiğin Kütle Spektrumu

1 Nolu Bileşiğin Kütle Spektrumu

1 Nolu Bileşiğin Kütle Spektrumu

1 Nolu Bileşiğin Kütle Spektrumu

1 Nolu Bileşiğin Kütle Spektrumu

1 Nolu Bileşiğin Kütle Spektrumu

1 Nolu Bileşiğin Kütle Spektrumu

1 Nolu Bileşiğin Kütle Spektrumu

1 Nolu Bileşiğin Kütle Spektrumu

1 Nolu Bileşiğin Kütle Spektrumu

1 Nolu Bileşiğin Kütle Spektrumu

1 Nolu Bileşiğin Kütle Spektrumu

1 Nolu Bileşiğin Kütle Spektrumu

1 Nolu Bileşiğin Kütle Spektrumu

1 Nolu Bileşiğin Kütle Spektrumu

1 Nolu Bileşiğin Kütle Spektrumu

1 Nolu Bileşiğin Kütle Spektrumu

1 Nolu Bileşiğin Kütle Spektrumu

1 Nolu Bileşiğin Kütle Spektrumu

1 Nolu Bileşiğin Kütle Spektrumu

1 Nolu Bileşiğin Kütle Spektrumu

1 Nolu Bileşiğin Kütle Spektrumu

1 Nolu Bileşiğin Kütle Spektrumu

1 Nolu Bileşiğin Kütle Spektrumu

1 Nolu Bileşiğin Kütle Spektrumu

1 Nolu Bileşiğin Kütle Spektrumu

1 Nolu Bileşiğin Kütle Spektrumu


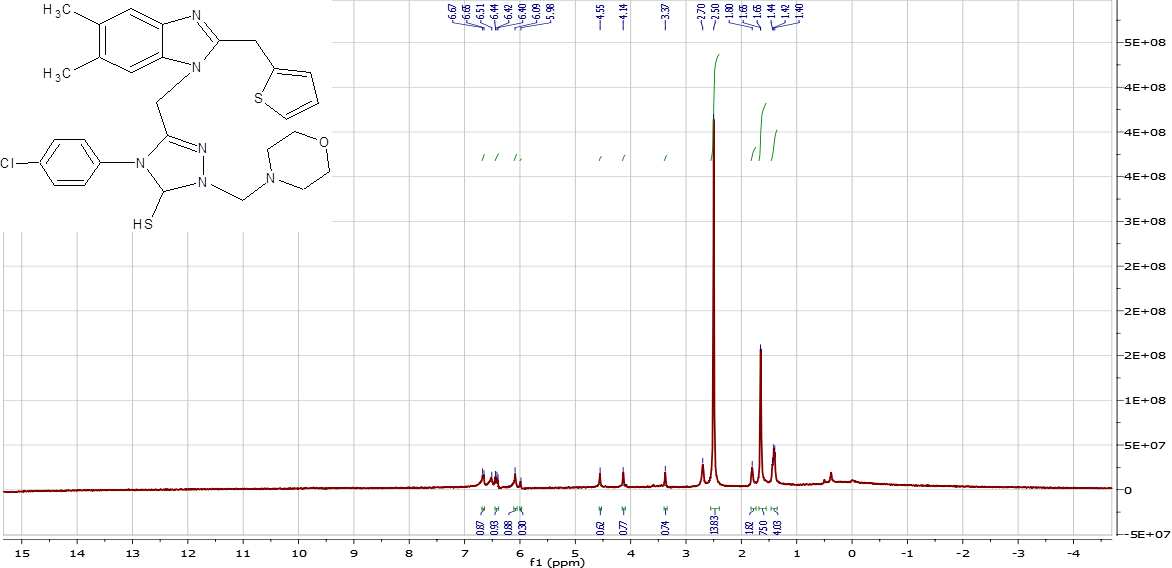


Compound7 ^1^H NMR Spectrum, 400 MHz, DMSO-d6


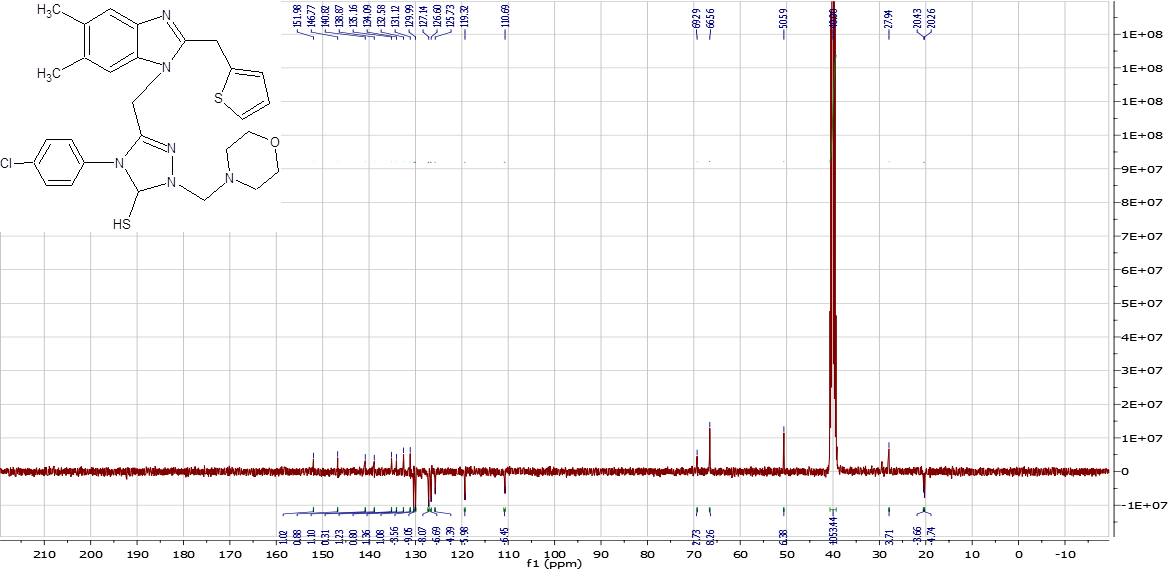


Compound 7 ^13^C APT NMR Spectrum, 100 MHz, DMSO-d6

1 Nolu Bileşiğin ^1^H NMR Spektrumu, 100 MHz, DMSO-d6

1 Nolu Bileşiğin ^1^H NMR Spektrumu, 100 MHz, DMSO-d6

1 Nolu Bileşiğin ^1^H NMR Spektrumu, 100 MHz, DMSO-d6

1 Nolu Bileşiğin ^1^H NMR Spektrumu, 100 MHz, DMSO-d6

1 Nolu Bileşiğin ^1^H NMR Spektrumu, 100 MHz, DMSO-d6

1 Nolu Bileşiğin ^1^H NMR Spektrumu, 100 MHz, DMSO-d6

1 Nolu Bileşiğin ^1^H NMR Spektrumu, 100 MHz, DMSO-d6

1 Nolu Bileşiğin ^1^H NMR Spektrumu, 100 MHz, DMSO-d6

1 Nolu Bileşiğin ^1^H NMR Spektrumu, 100 MHz, DMSO-d6

1 Nolu Bileşiğin ^1^H NMR Spektrumu, 100 MHz, DMSO-d6

1 Nolu Bileşiğin ^1^H NMR Spektrumu, 100 MHz, DMSO-d6

1 Nolu Bileşiğin ^1^H NMR Spektrumu, 100 MHz, DMSO-d6

1 Nolu Bileşiğin ^1^H NMR Spektrumu, 100 MHz, DMSO-d6

1 Nolu Bileşiğin ^1^H NMR Spektrumu, 100 MHz, DMSO-d6

1 Nolu Bileşiğin ^1^H NMR Spektrumu, 100 MHz, DMSO-d6

1 Nolu Bileşiğin ^1^H NMR Spektrumu, 100 MHz, DMSO-d6

1 Nolu Bileşiğin ^1^H NMR Spektrumu, 100 MHz, DMSO-d6

1 Nolu Bileşiğin ^1^H NMR Spektrumu, 100 MHz, DMSO-d6

1 Nolu Bileşiğin ^1^H NMR Spektrumu, 100 MHz, DMSO-d6

1 Nolu Bileşiğin ^1^H NMR Spektrumu, 100 MHz, DMSO-d6

1 Nolu Bileşiğin ^1^H NMR Spektrumu, 100 MHz, DMSO-d6

1 Nolu Bileşiğin ^1^H NMR Spektrumu, 100 MHz, DMSO-d6

1 Nolu Bileşiğin ^1^H NMR Spektrumu, 100 MHz, DMSO-d6

1 Nolu Bileşiğin ^1^H NMR Spektrumu, 100 MHz, DMSO-d6

1 Nolu Bileşiğin ^1^H NMR Spektrumu, 100 MHz, DMSO-d6

1 Nolu Bileşiğin ^1^H NMR Spektrumu, 100 MHz, DMSO-d6

1 Nolu Bileşiğin ^1^H NMR Spektrumu, 100 MHz, DMSO-d6

1 Nolu Bileşiğin ^1^H NMR Spektrumu, 100 MHz, DMSO-d6

1 Nolu Bileşiğin ^1^H NMR Spektrumu, 100 MHz, DMSO-d6

1 Nolu Bileşiğin ^1^H NMR Spektrumu, 100 MHz, DMSO-d6

1 Nolu Bileşiğin ^1^H NMR Spektrumu, 100 MHz, DMSO-d6


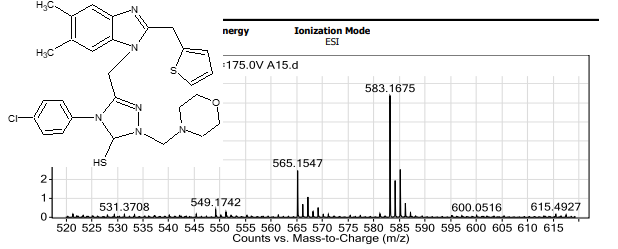


Compound 7 LC/TOF-MS

1 Nolu Bileşiğin Kütle Spektrumu

1 Nolu Bileşiğin Kütle Spektrumu

1 Nolu Bileşiğin Kütle Spektrumu

1 Nolu Bileşiğin Kütle Spektrumu

1 Nolu Bileşiğin Kütle Spektrumu

1 Nolu Bileşiğin Kütle Spektrumu

1 Nolu Bileşiğin Kütle Spektrumu

1 Nolu Bileşiğin Kütle Spektrumu

1 Nolu Bileşiğin Kütle Spektrumu

1 Nolu Bileşiğin Kütle Spektrumu

1 Nolu Bileşiğin Kütle Spektrumu

1 Nolu Bileşiğin Kütle Spektrumu

1 Nolu Bileşiğin Kütle Spektrumu

1 Nolu Bileşiğin Kütle Spektrumu

1 Nolu Bileşiğin Kütle Spektrumu

1 Nolu Bileşiğin Kütle Spektrumu

1 Nolu Bileşiğin Kütle Spektrumu

1 Nolu Bileşiğin Kütle Spektrumu

1 Nolu Bileşiğin Kütle Spektrumu

1 Nolu Bileşiğin Kütle Spektrumu

1 Nolu Bileşiğin Kütle Spektrumu

1 Nolu Bileşiğin Kütle Spektrumu

1 Nolu Bileşiğin Kütle Spektrumu

1 Nolu Bileşiğin Kütle Spektrumu

1 Nolu Bileşiğin Kütle Spektrumu

1 Nolu Bileşiğin Kütle Spektrumu

1 Nolu Bileşiğin Kütle Spektrumu

1 Nolu Bileşiğin Kütle Spektrumu

1 Nolu Bileşiğin Kütle Spektrumu

1 Nolu Bileşiğin Kütle Spektrumu

1 Nolu Bileşiğin Kütle Spektrumu


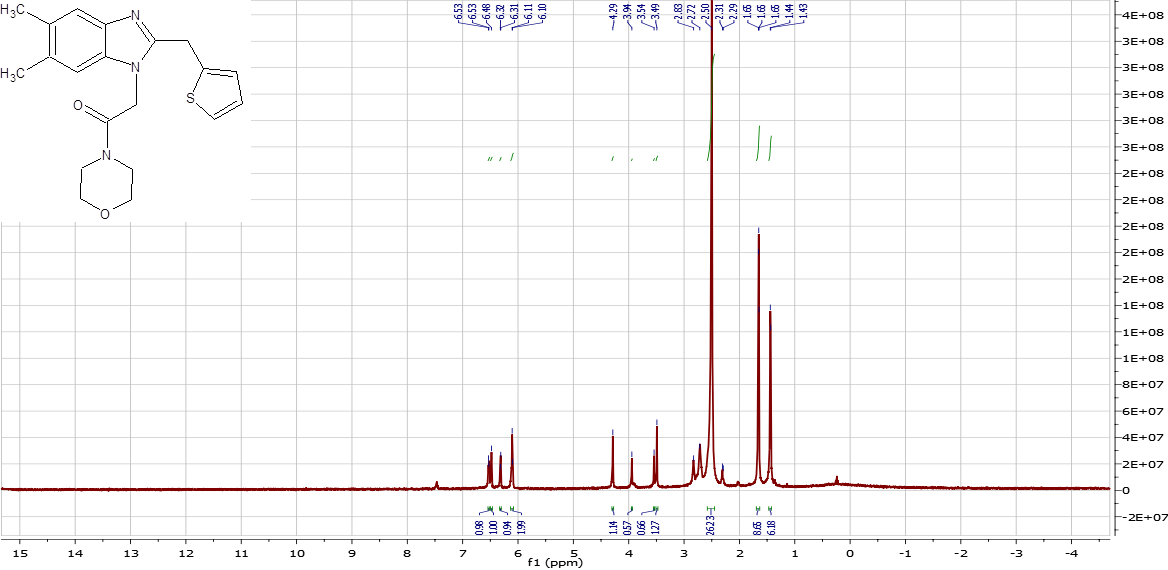


Compound 8a ^1^H NMR Spectrum, 400 MHz, DMSO-d6


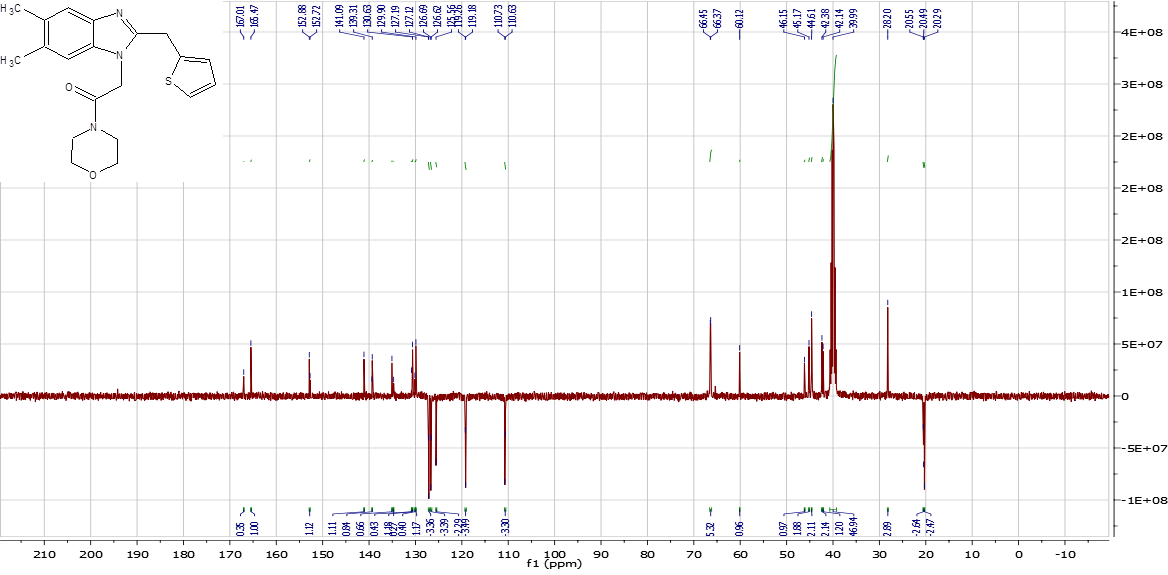


Compound 8a ^13^C APT NMR Spectrum, 100 MHz, DMSO-d6

1 Nolu Bileşiğin ^1^H NMR Spektrumu, 100 MHz, DMSO-d6

1 Nolu Bileşiğin ^1^H NMR Spektrumu, 100 MHz, DMSO-d6

1 Nolu Bileşiğin ^1^H NMR Spektrumu, 100 MHz, DMSO-d6

1 Nolu Bileşiğin ^1^H NMR Spektrumu, 100 MHz, DMSO-d6

1 Nolu Bileşiğin ^1^H NMR Spektrumu, 100 MHz, DMSO-d6

1 Nolu Bileşiğin ^1^H NMR Spektrumu, 100 MHz, DMSO-d6

1 Nolu Bileşiğin ^1^H NMR Spektrumu, 100 MHz, DMSO-d6

1 Nolu Bileşiğin ^1^H NMR Spektrumu, 100 MHz, DMSO-d6

1 Nolu Bileşiğin ^1^H NMR Spektrumu, 100 MHz, DMSO-d6

1 Nolu Bileşiğin ^1^H NMR Spektrumu, 100 MHz, DMSO-d6

1 Nolu Bileşiğin ^1^H NMR Spektrumu, 100 MHz, DMSO-d6

1 Nolu Bileşiğin ^1^H NMR Spektrumu, 100 MHz, DMSO-d6

1 Nolu Bileşiğin ^1^H NMR Spektrumu, 100 MHz, DMSO-d6

1 Nolu Bileşiğin ^1^H NMR Spektrumu, 100 MHz, DMSO-d6

1 Nolu Bileşiğin ^1^H NMR Spektrumu, 100 MHz, DMSO-d6

1 Nolu Bileşiğin ^1^H NMR Spektrumu, 100 MHz, DMSO-d6

1 Nolu Bileşiğin ^1^H NMR Spektrumu, 100 MHz, DMSO-d6

1 Nolu Bileşiğin ^1^H NMR Spektrumu, 100 MHz, DMSO-d6

1 Nolu Bileşiğin ^1^H NMR Spektrumu, 100 MHz, DMSO-d6

1 Nolu Bileşiğin ^1^H NMR Spektrumu, 100 MHz, DMSO-d6

1 Nolu Bileşiğin ^1^H NMR Spektrumu, 100 MHz, DMSO-d6

1 Nolu Bileşiğin ^1^H NMR Spektrumu, 100 MHz, DMSO-d6

1 Nolu Bileşiğin ^1^H NMR Spektrumu, 100 MHz, DMSO-d6

1 Nolu Bileşiğin ^1^H NMR Spektrumu, 100 MHz, DMSO-d6

1 Nolu Bileşiğin ^1^H NMR Spektrumu, 100 MHz, DMSO-d6

1 Nolu Bileşiğin ^1^H NMR Spektrumu, 100 MHz, DMSO-d6

1 Nolu Bileşiğin ^1^H NMR Spektrumu, 100 MHz, DMSO-d6

1 Nolu Bileşiğin ^1^H NMR Spektrumu, 100 MHz, DMSO-d6

1 Nolu Bileşiğin ^1^H NMR Spektrumu, 100 MHz, DMSO-d6

1 Nolu Bileşiğin ^1^H NMR Spektrumu, 100 MHz, DMSO-d6

1 Nolu Bileşiğin ^1^H NMR Spektrumu, 100 MHz, DMSO-d6


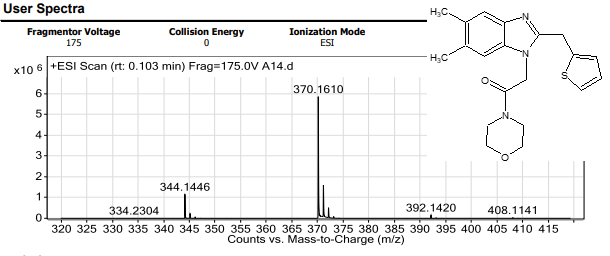


Compound 8a LC/TOF-MS

1 Nolu Bileşiğin Kütle Spektrumu

1 Nolu Bileşiğin Kütle Spektrumu

1 Nolu Bileşiğin Kütle Spektrumu

1 Nolu Bileşiğin Kütle Spektrumu

1 Nolu Bileşiğin Kütle Spektrumu

1 Nolu Bileşiğin Kütle Spektrumu

1 Nolu Bileşiğin Kütle Spektrumu

1 Nolu Bileşiğin Kütle Spektrumu

1 Nolu Bileşiğin Kütle Spektrumu

1 Nolu Bileşiğin Kütle Spektrumu

1 Nolu Bileşiğin Kütle Spektrumu

1 Nolu Bileşiğin Kütle Spektrumu

1 Nolu Bileşiğin Kütle Spektrumu

1 Nolu Bileşiğin Kütle Spektrumu

1 Nolu Bileşiğin Kütle Spektrumu

1 Nolu Bileşiğin Kütle Spektrumu

1 Nolu Bileşiğin Kütle Spektrumu

1 Nolu Bileşiğin Kütle Spektrumu

1 Nolu Bileşiğin Kütle Spektrumu

1 Nolu Bileşiğin Kütle Spektrumu

1 Nolu Bileşiğin Kütle Spektrumu

1 Nolu Bileşiğin Kütle Spektrumu

1 Nolu Bileşiğin Kütle Spektrumu

1 Nolu Bileşiğin Kütle Spektrumu

1 Nolu Bileşiğin Kütle Spektrumu

1 Nolu Bileşiğin Kütle Spektrumu

1 Nolu Bileşiğin Kütle Spektrumu

1 Nolu Bileşiğin Kütle Spektrumu

1 Nolu Bileşiğin Kütle Spektrumu

1 Nolu Bileşiğin Kütle Spektrumu

1 Nolu Bileşiğin Kütle Spektrumu


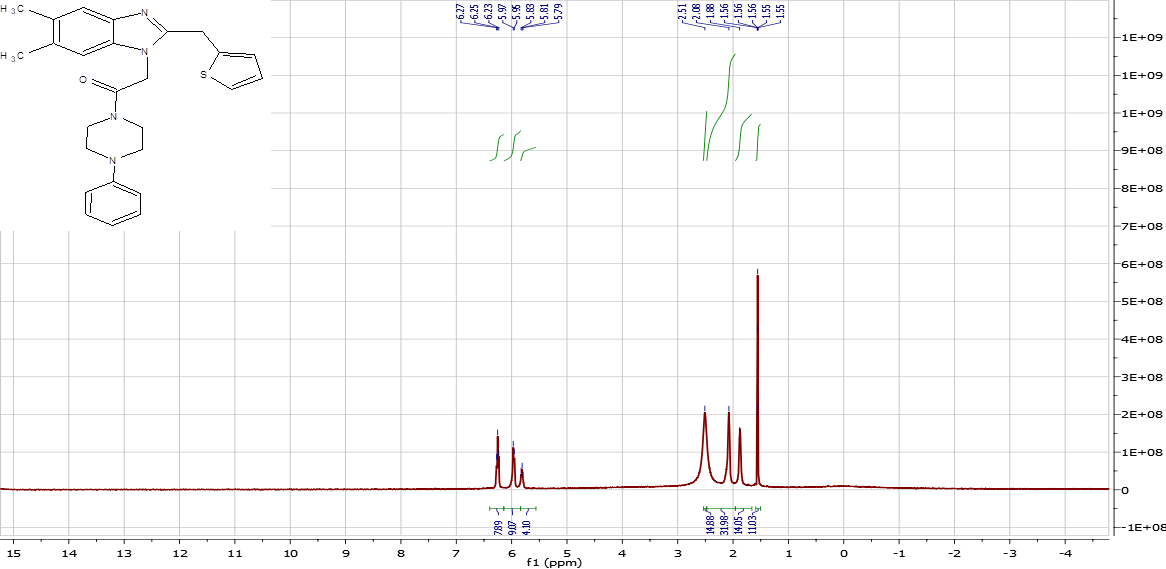


Compound 8b ^1^H NMR Spectrum, 400 MHz, DMSO-d6


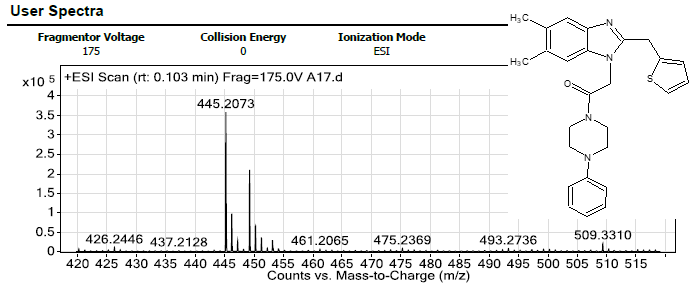


Compound 8b LC/TOF-MS

1 Nolu Bileşiğin Kütle Spektrumu

1 Nolu Bileşiğin Kütle Spektrumu

1 Nolu Bileşiğin Kütle Spektrumu

1 Nolu Bileşiğin Kütle Spektrumu

1 Nolu Bileşiğin Kütle Spektrumu

1 Nolu Bileşiğin Kütle Spektrumu

1 Nolu Bileşiğin Kütle Spektrumu

1 Nolu Bileşiğin Kütle Spektrumu

1 Nolu Bileşiğin Kütle Spektrumu

1 Nolu Bileşiğin Kütle Spektrumu

1 Nolu Bileşiğin Kütle Spektrumu

1 Nolu Bileşiğin Kütle Spektrumu

1 Nolu Bileşiğin Kütle Spektrumu

1 Nolu Bileşiğin Kütle Spektrumu

1 Nolu Bileşiğin Kütle Spektrumu

1 Nolu Bileşiğin Kütle Spektrumu

1 Nolu Bileşiğin Kütle Spektrumu

1 Nolu Bileşiğin Kütle Spektrumu

1 Nolu Bileşiğin Kütle Spektrumu

1 Nolu Bileşiğin Kütle Spektrumu

1 Nolu Bileşiğin Kütle Spektrumu

1 Nolu Bileşiğin Kütle Spektrumu

1 Nolu Bileşiğin Kütle Spektrumu

1 Nolu Bileşiğin Kütle Spektrumu

1 Nolu Bileşiğin Kütle Spektrumu

1 Nolu Bileşiğin Kütle Spektrumu

1 Nolu Bileşiğin Kütle Spektrumu

1 Nolu Bileşiğin Kütle Spektrumu

1 Nolu Bileşiğin Kütle Spektrumu

1 Nolu Bileşiğin Kütle Spektrumu

1 Nolu Bileşiğin Kütle Spektrumu
